# Genome-Wide Association Study Meta-Analysis of Central Serous Chorioretinopathy

**DOI:** 10.1001/jamaophthalmol.2026.4427

**Published:** 2026-11-05

**Authors:** Liyin Chen, Soo Hyun Francesca Kim, Buu Truong, Joel T. Rämö, Bryan R. Gorman, Elon H. C. van Dijk, Joost Brinks, Tiit Nikopensius, Seung Hoan Choi, Risto Kajanne, Juha Mehtonen, Kai Kaarniranta, Lucia Sobrin, Mitja Kurki, Suzanne Yzer, Wen-Chih Wu, Joni A. Turunen, Ayellet V. Segrè, Josep Maria Mercader, Alicia Huerta, Mark J. Daly, Aarno Palotie, Patrick T. Ellinor, Camiel J. F. Boon, Sudha K. Iyengar, Neal S. Peachey, Pradeep Natarajan, Elizabeth J. Rossin

**Affiliations:** 1 Harvard Medical School Department of Ophthalmology, Massachusetts Eye and Ear, Boston; 2 Broad Institute of MIT and Harvard, Cambridge, Massachusetts; 3 Heart and Vascular Institute and Center for Genomic Medicine, Mass General Brigham, Boston, Massachusetts; 4 Institute for Molecular Medicine Finland (FIMM), Helsinki Institute of Life Science (HiLIFE), University of Helsinki, Helsinki, Finland; 5 Massachusetts Eye and Ear, Boston; 6 Cardiovascular Disease Initiative, Broad Institute of MIT and Harvard, Cambridge, Massachusetts; 7 Center for Data and Computational Sciences (C-DACS), VA Boston Healthcare System, Boston, Massachusetts; 8 Booz Allen Hamilton, McLean, Virginia; 9 Department of Ophthalmology, Leiden University Medical Center, Leiden, the Netherlands; 10 Department of Vitreoretinal Surgery, Rotterdam Eye Hospital, Rotterdam, the Netherlands; 11 Estonian Genome Center, University of Tartu, Tartu, Estonia; 12 Department of Biostatistics, Boston University, Boston, Massachusetts; 13 Department of Ophthalmology, University of Eastern Finland and Kuopio University Hospital, Kuopio, Finland; 14 Program in Medical and Population Genetics, Broad Institute of MIT and Harvard, Cambridge, Massachusetts; 15 Department of Ophthalmology, Radboud University Medical Center, Nijmegen, the Netherlands; 16 Donders Institute for Brain, Cognition and Behaviour, Radboud University Medical Center, Nijmegen, the Netherlands; 17 Section of Cardiology, Medical Service, VA Providence Healthcare System, Providence, Rhode Island; 18 Eye Genetics Group, Folkhälsan Research Center, Helsinki, Finland; 19 Department of Ophthalmology, University of Helsinki and Helsinki University Hospital, Helsinki, Finland; 20 Diabetes Unit, Massachusetts General Hospital, Boston; 21 Programs in Metabolism and Medical and Population Genetics, Broad Institute of MIT and Harvard, Cambridge, Massachusetts; 22 Center for Genomic Medicine, Massachusetts General Hospital, Boston; 23 Department of Medicine, Harvard Medical School, Boston, Massachusetts; 24 Novo Nordisk Foundation Center for Genomic Mechanisms of Disease, Broad Institute of MIT and Harvard, Cambridge, Massachusetts; 25 Analytic and Translational Genetics Unit, Massachusetts General Hospital and Harvard Medical School, Boston; 26 Stanley Center for Psychiatric Research, Broad Institute of MIT and Harvard, Cambridge, Massachusetts; 27 Psychiatric and Neurodevelopmental Genetics Unit, Massachusetts General Hospital and Harvard Medical School, Boston; 28 Department of Neurology, Massachusetts General Hospital, Boston; 29 Analytic and Translational Genetics Unit, Massachusetts General Hospital and Harvard Medical School, Boston; 30 Cardiovascular Research Center, Mass General Brigham, Harvard Medical School, Boston; 31 Department of Ophthalmology, Amsterdam University Medical Center, University of Amsterdam, Amsterdam, the Netherlands; 32 Cleveland Institute for Computational Biology, Case Western Reserve University, Cleveland, Ohio; 33 Department of Population and Quantitative Health Sciences, Case Western Reserve University School of Medicine, Cleveland, Ohio; 34 Research Service, VA Northeast Ohio Healthcare System, Cleveland; 35 Cole Eye Institute, Cleveland Clinic Foundation, Cleveland, Ohio; 36 Department of Ophthalmology, Cleveland Clinic Lerner College of Medicine of Case Western Reserve University, Cleveland, Ohio; 37 Amgen, Thousand Oaks, California

## Abstract

**Importance:**

The pathophysiology of central serous chorioretinopathy (CSC) remains incompletely understood, and there is ongoing debate regarding the origin of primary dysfunction.

**Objective:**

To elucidate the genetic architecture of CSC and map susceptibility loci to specific ocular cell types and molecular pathways.

**Design, Setting, and Participants:**

This genetic association study was a genome-wide association study (GWAS) meta-analysis aggregating data from 5 large biobank cohorts and included 2584 patients with CSC and 1 044 455 control participants of European ancestry. Cohort data were from July 1, 2010, to October 31, 2023; data analysis was performed from Febuary 2024 to January 2026. Post-GWAS functional mapping was performed using mendelian randomization, bayesian colocalization, and single-cell disease relevance scoring across human ocular tissue atlases. The relevance of the genetic findings was demonstrated by building a CSC genetic risk score and applying it to the UK Biobank (UKB).

**Main Outcomes and Measures:**

Identification of risk loci reaching the genome-wide threshold, prioritized putative causal genes, enrichment of genetic risk within specific ocular tissues and cell types, and performance of a GRS in the independent UKB dataset.

**Results:**

The meta-analysis identified 10 risk loci reaching the genome-wide threshold (*P* < 5 × 10^−8^), including 3 novel loci (*TGFB1*, *LINC00551*, and *LOC105375630*) and 7 replicated loci (*CFH*, *GATA5*, *PREX1*, *CD46*, *NOTCH4, PTPRB,* and *TNFRSF10A*). Integrative post-GWAS analysis prioritized 10 genes, including *TGFB1*, *TNFRSF10A*, and *CFH,* as likely causal effectors. Single-cell enrichment mapping revealed that CSC genetic risk is concentrated in scleral tissue (*P* < .001) and vascular endothelial cells (*P* < .001). Mendelian randomization pointed to circulating TGFB1 and TNFRSF10A levels as associated with CSC risk. A CSC genetic risk score applied in UKB revealed a higher frequency of CSC-relevant abnormalities on macular optical coherence tomography in participants in the top 1% of polygenic risk (18/109 [16.5%] vs 8/134 [6.0%]; difference, 10.5 percentage points; 95% CI, 2.6-19.1; adjusted odds ratio, 4.05; 95% CI, 1.65-10.87; *P* = .002).

**Conclusions and Relevance:**

Convergent genetic evidence supports CSC as a primary disorder of the sclera and choroidal vasculature with retinal pigment epithelium changes likely representing a downstream consequence. Integrative post-GWAS analysis pointed to complement regulation (*CFH, CD46*), vascular integrity and endothelial cell signaling (*PTPRB, TNFRSF10A, NOTCH4, PREX1*), and scleral extracellular matrix biology (*TGFB1*) as important in CSC.

## Key Points

**Question:**

What genetic loci, genes, proteins, and ocular cell types underlie susceptibility to central serous chorioretinopathy (CSC)?

**Findings:**

In this genetic association study including 2584 CSC cases and 1 044 455 controls, 10 loci passing a genome-wide threshold were identified, including 3 novel loci. Integrative analyses implicated scleral fibroblasts and vascular endothelial cells, prioritized candidate effector genes and circulating proteins, and showed that high CSC genetic risk was associated with more frequent retinal pigment epithelium abnormalities on macular optical coherence tomography.

**Meaning:**

These findings support CSC as a primary sclerovascular disorder and nominate mechanism-linked pathways for future translational studies.

## Teaser

This genetic association study analyzes data from 5 large biobank cohorts to determine the genetic loci, genes, proteins, and ocular cell types that underlie susceptibility to central serous chorioretinopathy.

## Introduction

Central serous chorioretinopathy (CSC) is an important cause of central vision loss in working-aged adults, but its pathophysiology remains incompletely understood. Symptoms can involve metamorphopsia, decreased visual acuity, and central scotoma.^1^ The age of onset of CSC is between 20 and 50 years, and males are more commonly affected by up to 6-fold.^2^ It remains a multifactorial disease with associated risk factors, including corticosteroid use, type A personality, psychological stress, obstructive sleep apnea, *Helicobacter pylori* infection, uncontrolled hypertension, and pregnancy. At the anatomic level, CSC is associated with abnormally dilated and leaking choroidal vasculature, first characterized by indocyanine green angiography^4^; retinal pigment epithelium (RPE) dysfunction and disruption; and abnormally thickened sclera,^8^ with increased anterior scleral thickness positively correlating with subfoveal choroidal thickening.^9^

Recent advances in population genetics have offered novel insights into the etiology of CSC. Roughly half of patients with CSC have at least 1 relative also present with CSC symptoms, suggesting a genetic predisposition.^11^ Genome-wide association studies (GWAS) in European^13^ and Japanese^14^ patients identified loci near complement factor H (*CFH*), *GATA5*, and *TNFRSF10A*. We previously published a 3-cohort meta-analysis of CSC that identified novel loci near *PREX1*, *CD46*, and *NOTCH4*, pointing to the likely role of endothelial dysfunction.^16^ Following this, a large single-variant focused meta-analysis showed a strong association with the specific missense variant rs113791087 in *PTPRB* that co-associates with varicose veins.^17^ A recent meta-analysis of Japanese and European cohorts confirmed involvement of *CFH*, *TNFRSF10A*, and *GATA5* but was unable to replicate other loci.^18^ Overall, studies are still underpowered, and most have limited post-GWAS analyses to nominate causal genes and cell types.

To address these limitations, we performed a European CSC case-control GWAS meta-analysis with an integrated post-GWAS framework tailored to CSC biology. We aggregated 5 independent cohorts and deployed complementary post-GWAS analyses to test whether genetically proxied circulating protein changes are associated with CSC. Single-cell disease-relevance scoring across ocular atlases then localized genetic risk to discrete ocular cell types and tissues, and a genetic-risk-to-ocular-anatomy bridge in the UK Biobank (UKB) optical coherence tomography (OCT) data evaluated whether polygenic burden manifests as measurable alterations on macular OCT.

## Methods

### Study Design

We conducted a GWAS meta-analysis of CSC using 5 European ancestry cohorts: FinnGen,^19^ All of Us research program,^20^ Mass General Brigham Biobank (MGBB),^21^ Million Veteran Program (MVP),^23^ and a previously published Dutch cohort.^13^ Cohort data were from July 1, 2010, to October 31, 2023; data analysis was performed from Febuary 2024 to January 2026. This study is reported in accordance with the Strengthening the Reporting of Genetic Association Studies (STREGA) reporting guideline, an extension of the Strengthening the Reporting of Observational Studies in Epidemiology (STROBE) statement. Details regarding the study cohorts are in eMethods 1 in Supplement 1.

The MGBB study protocol was approved by the MGB institutional review board. Use of All of Us data for this study was approved by the All of Us research program after completing all required training. The study of European patients with CSC was carried out in accordance with the tenets of the Declaration of Helsinki and was approved by the local ethics committees of the Radboud University Medical Center, Leiden University Medical Center, and University Hospital of Cologne. Written informed consent was obtained for all MGBB, All of Us, and Dutch participants with CSC. The MVP024 study protocol was approved by the Veterans Affairs central institutional review board. The complete ethics statement of the FinnGen Study is in eMethods 2 in Supplement 1.

### Phenotype Ascertainment

CSC was defined using cohort-specific electronic health record codes or previously published phenotype definitions. Detailed case-control definitions are provided in eMethods 3 in Supplement 1.

### Genome-Wide Association Studies and Meta-Analysis

The GWAS in FinnGen, All of Us, MVP, and MGBB were performed using REGENIE (version 3; version 2.2.4 for FinnGen),^24^ limiting to European samples (ancestry definitions for each cohort are described eMethods 1 in Supplement 1). The GWAS for the Dutch cohort was described previously.^13^ All analyses were adjusted for age, age squared, sex, genotyping array/batch, and the first 10 genomic PCs. Genomic control inflation (λ_GC_) was computed to assess systemic confounding in each cohort. Quality control of the genotype data is described in eMethods 4 in Supplement 1.

We combined cohort-level summary statistics using the genome-wide association meta-analysis tool GWAMA (version 2.2.2).^25^ Variants were included if present in at least 2 cohorts. A total of 11 068 938 rsID-labeled single nucleotide variations (SNVs) were used for subsequent analyses. Locus definition followed a ±500-kb window around each lead SNV with linkage disequilibrium *r^2^* = 0.05 or greater using 1000 Genomes Phase 3 ^26^ European samples as a linkage disequilibrium reference.

Because case ascertainment in the Dutch chronic CSC cohort differed from the 4 cohorts based on *International Classification of Diseases* codes, we repeated the fixed-effects meta-analysis excluding the Dutch data (2063 cases; eMethods 5 in Supplement 1).

### Post-GWAS Analyses

The details of MAGMA, Polygenic Priority Score (PoPS), colocalization analyses (coloc and coloc.susie), mendelian randomization, and single-cell disease relevance score analysis (scDRS) can be found in eMethods 6 through 10 in Supplement 1 and followed standard default protocols. No adjustment was made for multiple comparisons across the full set of analyses; reported *P* values are nominal but adhere to a Bonferroni-adjusted threshold, and findings from the secondary analyses should be interpreted as exploratory.

### Genetic Risk Score and OCT in UKB

We constructed a CSC genetic risk score (GRS) using the 10 lead variants identified in the CSC meta-analysis (Table), weighted by meta-analysis log-odds ratios. Participants in the top and bottom 1% of the GRS distribution were selected for imaging review by a retinal specialist. Participant numbers at each selection step, quality exclusions, the rationale for the 1% threshold, and the full grading definition are provided in eMethods 11 in Supplement 1.

**Table.** Ten Loci Reaching the Genome-Wide Threshold in the CSC GWAS Meta-Analysis^a^

rsID	Nearest gene	CHR	Position^b^	A1	β^c^	SE	*P* value^d^	*P* value for heterogeneity^e^
rs10922105	*CFH*	1	196721120	C	0.349	0.0281	3.31 × 10^−35^	.10
rs2724360	*CD46*	1	207769813	T	0.297	0.0367	6.46 × 10^−16^	.56
rs113791087	*PTPRB*	12	70559589	G	1.109	0.141	4.82 × 10^−15^	.28
rs8192569	*NOTCH4*	6	32222707	A	0.319	0.0467	9.09 × 10^−12^	.40
rs2427357	*GATA5*	20	62450103	C	0.221	0.0325	1.20 × 10^−11^	.26
rs73045269^f^	*TGFB1*, *CCDC97*	19	41319286	T	−0.264	0.0402	5.02 × 10^−11^	.55
rs34822542	*PREX1*	20	48641217	C	0.2407	0.0401	2.04 × 10^−9^	.28
rs13278062	*TNFRSF10A*	8	23225458	T	0.165	0.0286	9.17 × 10^−9^	.31
rs150013629^f^	*LOC105375630*	8	88447463	G	1.197	0.2182	4.17 × 10^−8^	.23
rs1888541^f^	*LINC00551*	13	106697763	A	0.219	0.0400	4.53 × 10^−8^	.89

Abbreviations: A1, effect allele; CHR, chromosome; CSC, central serous chorioretinopathy; GWAS, genome-wide association studies; rsID, reference single nucleotide variation cluster identifier.

^a^ Results derived from a fixed-effects inverse-variance-weighted meta-analysis of 5 European-ancestry cohorts. Each cohort-level GWAS was performed with adjustment for age, age^2^, sex, genotyping array/batch, and the first 10 genomic principal components. Variants with *P* < 5 × 10^−8^ and present in ≥2 cohorts are shown.

^b^ Genomic position in GRCh38 coordinates (base pairs).

^c^ Per-allele log-odds ratio.

^d^ 2-Sided *P* value from the fixed-effects meta-analysis.

^e^ *P* value for Cochran *Q* test of between-study heterogeneity.

^f^ Novel loci not previously reported in CSC.

Macular spectral-domain OCT scans (Topcon 3D OCT-1000) from the selected high- and low-risk participants underwent manual grading by an attending retina specialist. The grader (E.J.R.) and analyst (L.C.) were masked to the participants’ GRS status. The primary outcome was the presence of any abnormalities seen in CSC in either eye including RPE detachment or other RPE irregularities, evidence of pattern dystrophy or pattern-like changes, subretinal fluid, evidence of CSC, or outer retinal atrophy. Unrelated findings like epiretinal membrane, drusen, or other subtle abnormalities were not counted. Scans were binarized as “abnormal” or “normal” based on the presence of any of the aforesaid findings. Differences in the proportion of participants with OCT abnormalities between GRS percentiles were assessed by multivariable logistic regression adjusted for age, sex, and the first 10 genomic principal components, with the likelihood-ratio test as the primary inference.

### Effect Size Comparison Between CSC and Age-Related Macular Degeneration

Because the CSC GWAS is still relatively underpowered for formal genetic correlation analysis, we compared effect sizes at CSC loci identified here and previously reported age-related macular degeneration (AMD) loci.^27^ For each locus, the lead variant was used when available in both datasets; otherwise, a proxy variant in linkage disequilibrium was chosen. Details regarding proxy variant selection are in eMethods 12 in Supplement 1. Pearson correlation coefficients were computed to quantify cross-trait concordance.

### Statistical Analysis

Significance thresholds were specified a priori. Genome-wide significance was set at *P* < 5 × 10^−8^, and between-cohort heterogeneity was assessed with the Cochran *Q* test at *P* < .10. Gene-based tests used a Bonferroni-corrected α of .05/17 832 and mendelian randomization estimates an α of .05/3 across the 3 colocalized proteins. Strong colocalization required a posterior probability of a shared causal variant (PP4) of 0.90 or greater together with a PP4/PP3 ratio above 3, and cell type and tissue enrichment required a Benjamini-Hochberg false discovery rate (FDR) below .05 within each dataset and grouping variable. All other comparisons used α = .05. Hypothesis tests were 2-sided, with the exception of the MAGMA and the scDRS tests, which are 1-sided by construction. Analyses were performed with MAGMA version 1.10, PoPS version 0.2, scDRS version 1.0.4, PLINK versions 1.9 and 2.0, and R version 4.4.3 with the coloc (version 5.2.3), susieR (version 0.12.35), and TwoSampleMR (version 0.6.15) extension packages.

## Results

### Meta-Analysis and Cohort-Level GWAS

Across the 5 cohorts, we analyzed 2584 CSC cases and 1 044 455 controls from European ancestry (eTables 1 and 2 in Supplement 2). A total of 11 068 938 variants were included for meta-analysis after quality control. Fixed-effects meta-analysis yielded 10 loci reaching the genome-wide significance threshold (Figure 1 and Table). Seven loci confirmed previous associations: variants near *CFH, CD46, NOTCH4, PREX1, PTPRB* and *GATA5*. We replicated a variant near *TNFRSF10A* for the first time in a European cohort, replicating a risk locus that was originally identified only in Japanese cohorts. Three novel genome-wide significant loci (*P* < 5 × 10^−8^) were identified, with the nearest genes *TGFB1, LINC00551*, and *LOC105375630*. Effect sizes and directions were similar for each locus in each cohort (Figure 2 and eTable 3 in Supplement 2) (*P* value for Cochran *Q* test statistic for heterogeneity ≥.10). The quantile-quantile plot showed modest deviation from the null consistent with polygenicity (λ_GC_ = 1.055) (eFigures 1 and 2 in Supplement 1).

**Figure 1.**
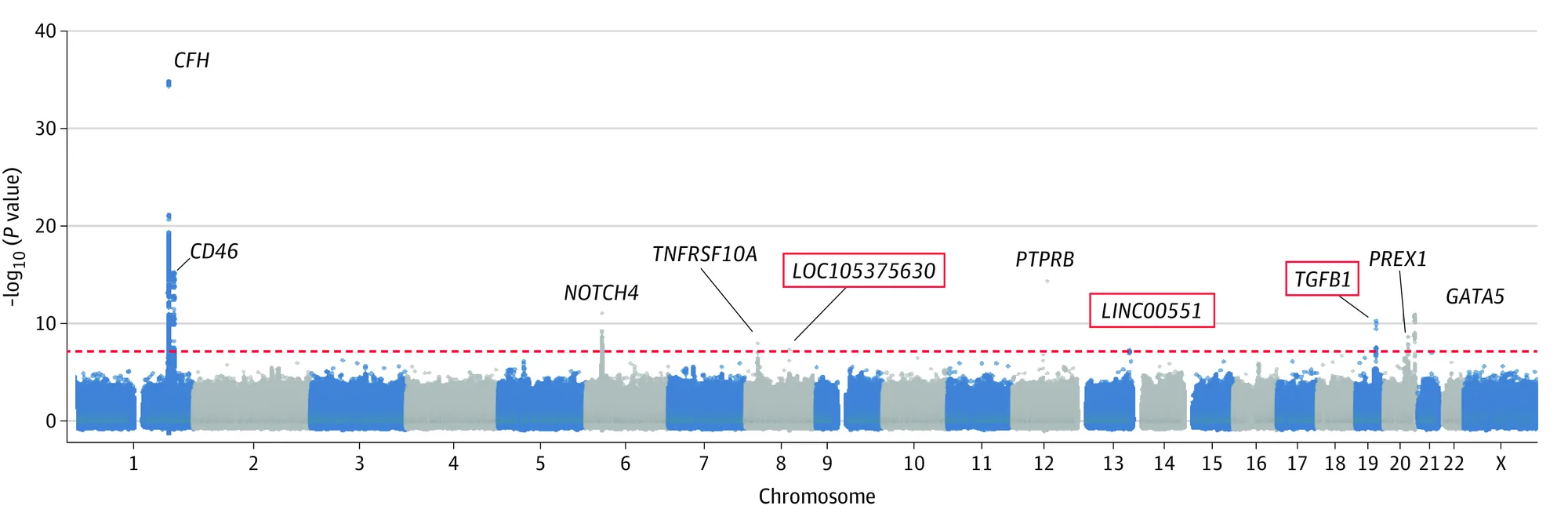
Graph Showing Identification of 10 Associated Loci From Genome-Wide Association Meta-Analysis of Central Serous Chorioretinopathy Manhattan plot of −log_10_(*P*) and chromosomal position for all variants present in the meta-analysis. Each point represents a single variant; chromosomes alternate in color. The horizontal red line indicates the genome-wide threshold (*P* = 5 × 10^−8^). The nearest gene is annotated for each locus reaching this threshold; novel loci are labeled in red boxes.

**Figure 2.**
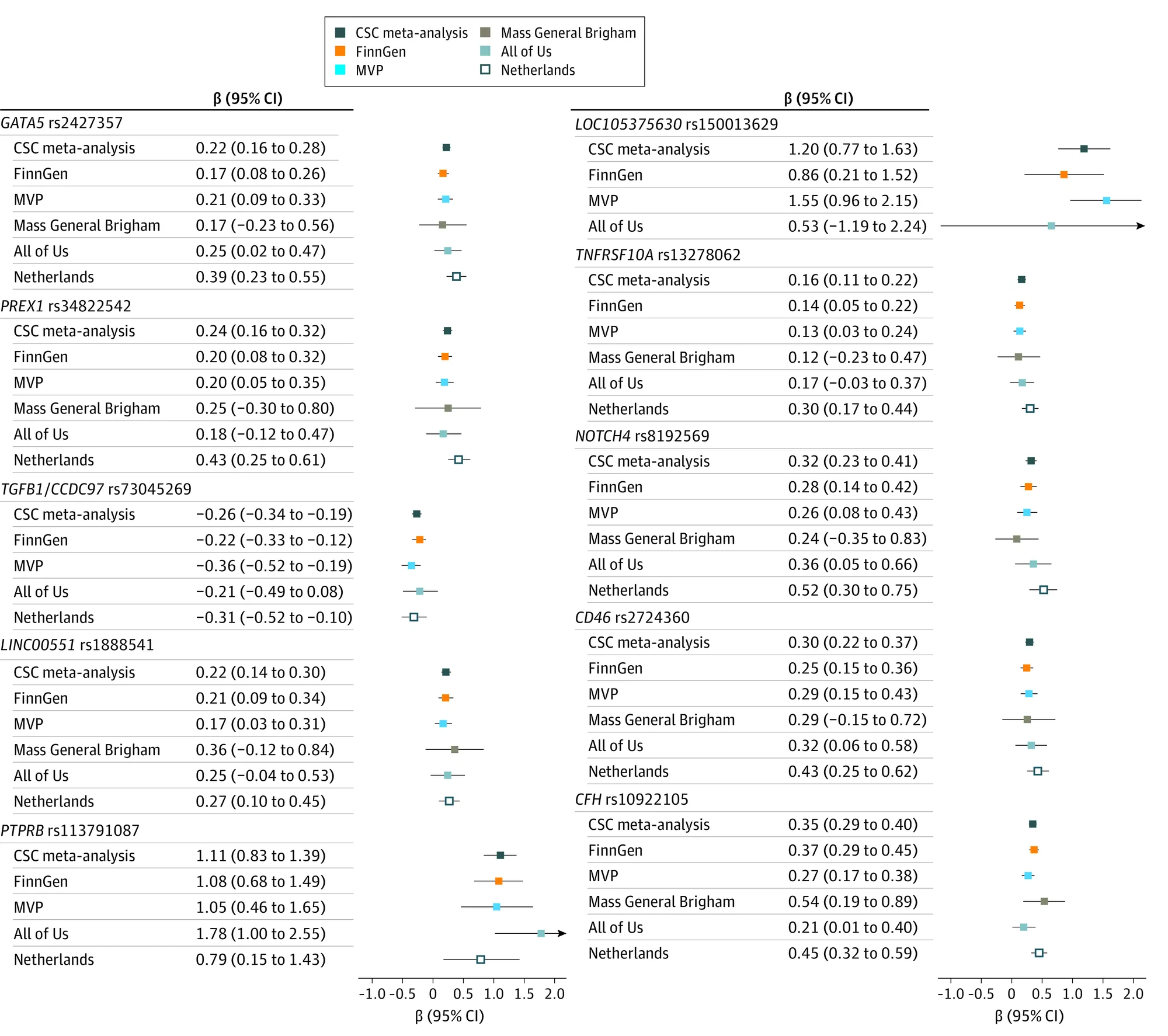
Forest Plots of Per-Allele Effect Sizes (Log-Odds Ratios) for the 10 Lead Variants in the Individual Cohort Genome-Wide Association Studies and the Meta-Analysis Corresponding per-variant summary statistics for the 10 lead variants are provided in eTable 3 in Supplement 2. CSC indicates central serous chorioretinopathy; MVP, VA Million Veteran Program.

In a sensitivity analysis excluding the Dutch chronic CSC cohort, all 10 lead variants retained the same direction of effect and 7 of 10 loci retained *P* < 5 × 10^−8^, consistent with the power reduction associated with a 20% reduction in case count (eTable 4 in Supplement 2 and eFigure 3 in Supplement 1).

### Gene Prioritization

We performed gene-based association analysis using MAGMA. Nineteen genes passed the Bonferroni-corrected threshold, among which 4 of them (*CD46, GATA5, PREX1*, and *CFH*) were nearest to the identified lead genome-wide SNV (eTable 5 in Supplement 2).

We applied the PoPS framework to propose effector genes with biological relevance. Of 10 GWAS loci, all but 1 locus had at least 1 positively scored gene from PoPS (eTable 6 in Supplement 2). PoPS prioritized genes agreed with the nearest-gene assignment for 7 of 10 loci, including *CFH, TNFRSF10A*, and *TGFB1*.

### Colocalization and Mendelian Randomization With Serum Protein Levels

To test whether any CSC variants modulate CSC risk through serum protein levels, we performed bayesian colocalization with 2923 serum proteins using the protein quantitative trait loci (pQTL) dataset from the UKB.^28^ We then carried out 2-sample mendelian randomization restricted to the proteins with strong colocalization evidence to test the direction and magnitude of the genetically proxied effect.

We identified strong evidence of colocalization (PP4 value >0.90) between the CSC GWAS signal and the pQTL signals for TNFRSF10A, TGFB1, and CASP10 (PP4 > 0.95 for all 3) (eTable 7 in Supplement 2). LocusCompare visualizations confirmed that the same high–linkage disequilibrium variant clusters drove both the pQTL and CSC GWAS signals for these proteins, verifying that the colocalization results reflect shared genetic architecture (eFigure 4A in Supplement 1). To allow for multiple causal variants per region, we also performed sum of single effects (SuSiE), and TGFB1 and TNFRSF10A again showed strong support for a shared causal variant (posterior probability, 0.99 and 1.00, respectively) (eTable 8 in Supplement 2). CASP10 colocalized under coloc but could not be assessed by SuSiE because its CSC signal did not form a credible set due to high linkage disequilibrium among 4 variants in the associated region.

We performed mendelian randomization to test whether altered serum protein levels contribute to CSC risk for the 3 proteins related to the association signals that colocalized. Among the 3, mendelian randomization estimates were directionally consistent across all complementary estimators. Genetically predicted higher circulating TNFRSF10A was associated with reduced CSC risk and passed the Bonferroni-corrected threshold of *P* < .05/3 = 0.017 (eFigure 4B in Supplement 1). TGFB1 and CASP10 also passed this threshold, with genetically predicted higher levels of each associated with lower CSC risk.

Effect-size estimates for all 3 proteins were concordant in direction and similar in magnitude across complementary mendelian randomization estimators (inverse-variance–weighted, simple median, and weighted median, mendelian randomization–Egger), with no evidence of directional horizontal pleiotropy (mendelian randomization–Egger intercept *P* > .05) (eTable 9 in Supplement 2).

### Endothelial and Scleral Enrichment in scRNA-Seq Data

To map CSC genetic risk to specific cell types, we applied scDRS to 3 complementary human single-cell atlases using a combined set of genes prioritized from the genetic analyses discussed above (eTable 10 in Supplement 2).

In the posterior segment atlas, scleral tissue achieved the highest score of all tested tissues (*P* < .001; *z* = 4.87; FDR <.05) (Figure 3).^29^ This finding was replicated in the multi-organ Tabula Sapiens atlas^31^ (*P* = .004; *z* = 3.70; FDR < .05). Additionally, the scleral lamina cribrosa, the scleral structure of the optic nerve head, demonstrated nominal enrichment (*P* = .04; *z* = 2.00; FDR < .10). Of note, vascular tissue is not a separate tissue category because it is not separated mechanically from other tissues.

**Figure 3.**
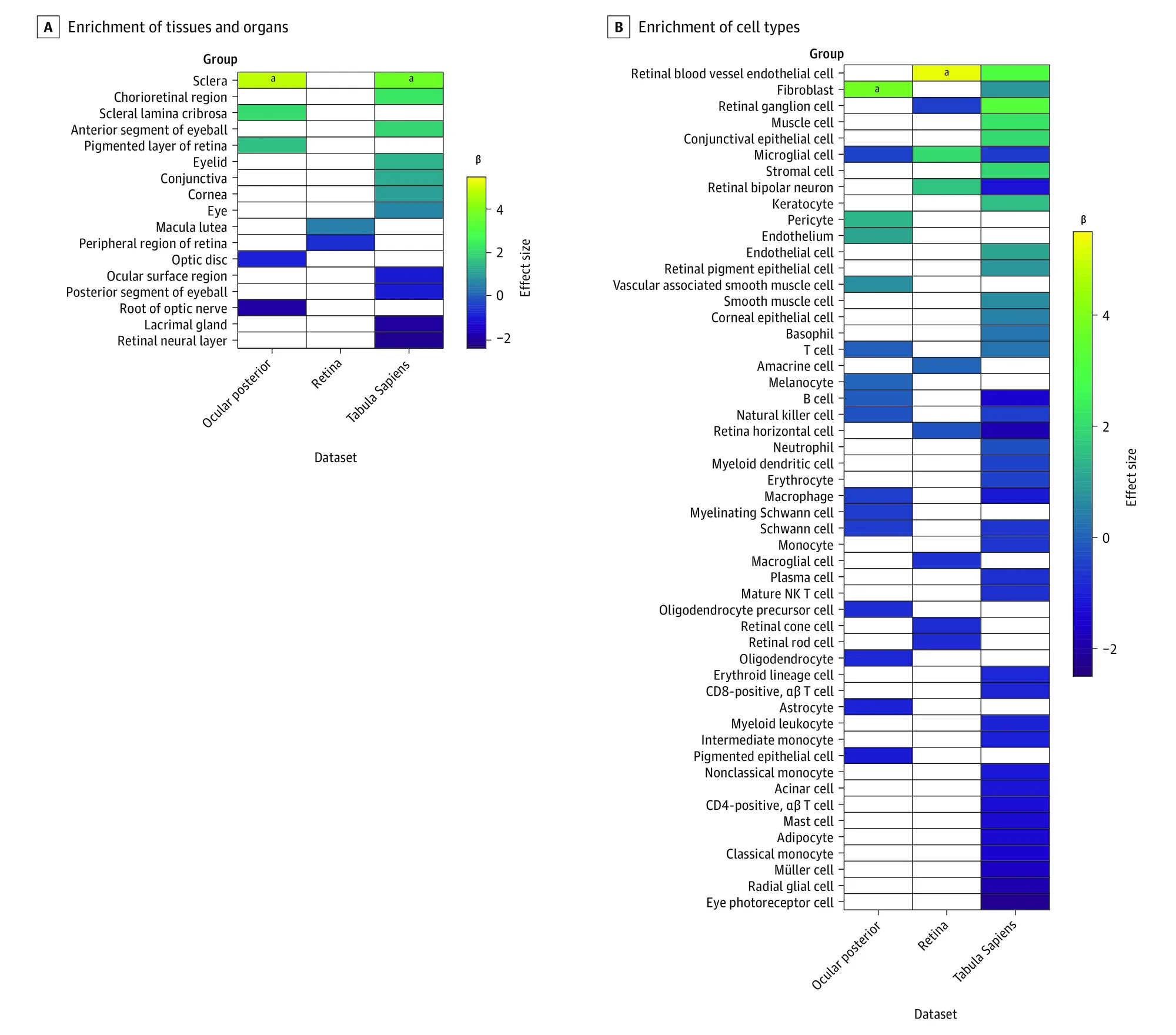
Heatmap Showing How Single-Cell Disease Relevance Scoring (scDRS) Revealed Vascular and Scleral Enrichment of Central Serous Chorioretinopathy (CSC) Genetic Risk Monte Carlo *z* scores from scDRS analysis of CSC genome-wide association studies (GWAS) summary statistics across anatomical regions and tissue types (A) and cell types (B). Columns represent 3 independent datasets: Monavarfeshani et al (posterior segment atlas),^29^ Menon et al (retinal atlas),^30^ and Tabula Sapiens (multi-organ atlas).^31^ Rows indicate anatomical groupings and tissue types. Color intensity represents the strength and direction of genetic association, with warm colors (yellow-green) indicating positive enrichment of CSC genetic risk and cool colors (purple-blue) indicating depletion. ^a^False discovery rate <.05.

At the single-cell level (whereby tissues are dissociated and homogenized into single cells), vascular endothelial cells and fibroblasts were identified as cellular expressors of CSC risk genes among all cell types in the 2 single-cell datasets containing these cells. In the specialized retinal atlas, retinal blood vessel endothelial cells were enriched (*P* < .001; *z* = 5.31; FDR < .05). This was corroborated by the Tabula Sapiens atlas, where retinal blood vessel endothelial cells (*P* = .006; *z* = 3.01; FDR = .11) exhibited the most substantial enrichment signal among the 52 cell types in the dataset. Fibroblasts, a key structural component of the sclera, displayed enrichment in the posterior segment atlas (*P* < .001; *z* = 3.81; FDR < .05) (eFigure 5 in Supplement 1).

In contrast to the robust vascular and scleral signals, cell types intrinsic to the neurosensory retina, immune system, and RPE showed no evidence of expression enrichment of genes associated to CSC. Furthermore, T cells, B cells, macrophages, and neutrophils consistently yielded null scores across all datasets (*P* > .05 for all immune subsets).

### CSC GRS Associated With RPE Abnormality in UKB

To evaluate whether polygenic burden for CSC manifests as measurable anatomic changes in an unselected population, we constructed a CSC GRS from the independent lead variants reaching the genome-wide threshold and applied it to unrelated European-ancestry UKB participants with macular OCT imaging. The proportion of participants with at least 1 CSC-related abnormality in either eye was 2.75-fold higher in the high-risk group (top 1% of GRS) than in the low-risk group (bottom 1% of GRS, 16.51% [18/109] vs 5.97% [8/134]). In multivariable logistic regression adjusted for age, sex, and the first 10 genomic principal components, high GRS status was associated with a nearly 4-fold increase in the odds of an abnormal OCT (odds ratio, 4.05; 95% CI, 1.65-10.87; likelihood-ratio *P* = .002). Representative high-GRS scans are shown in Figure 4.

**Figure 4.**
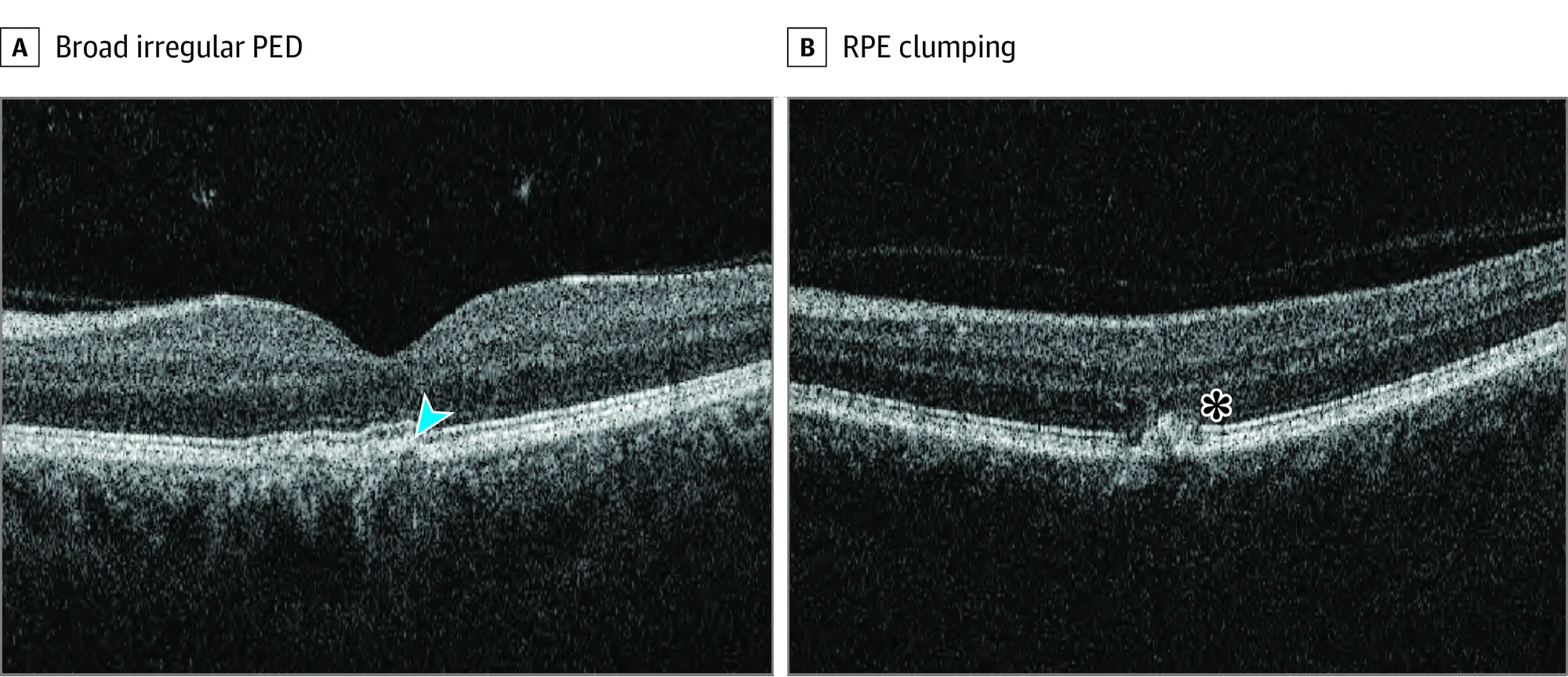
Representative Abnormal Macular Optical Coherence Tomography Scans From 2 UK Biobank Participants in the Top 1% of the Central Serous Chorioretinopathy Genetic Risk Score Distribution A, Broad irregular pigment epithelial detachment underneath the fovea (arrowhead). B, Retinal pigment epithelium clumping (asterisk). Images reproduced by kind permission of UK Biobank.

### Effect Size Comparison Between CSC and AMD

At established AMD risk loci, allelic effects for AMD and CSC were inversely correlated (*R* = −0.78; *P* = .004) (eFigure 6 in Supplement 1). A reciprocal analysis of CSC loci showed a similar direction of effect (*R* = −0.41; *P* = .49). These results were consistent with a prior report^16^ which found that while CSC and AMD partially share susceptibility loci, the direction of effect is frequently opposite.

## Discussion

This study leverages data from 5 cohorts and 1 047 039 individuals to identify novel risk loci and is paired with an integrative post-GWAS framework. Our results converged on a model that CSC susceptibility is concentrated in scleral fibroblasts and vascular endothelial cells. While RPE abnormalities are a clinical hallmark of CSC, these results support the classification of CSC as a disorder stemming first from the sclera and choroidal vasculature with RPE damage as a downstream consequence. The involvement of the choroid and sclera in CSC is itself well established from imaging studies, including choroidal vascular hyperpermeability on indocyanine green angiography,^6^ a thickened choroid on enhanced-depth-imaging OCT,^33^ and increased scleral thickness.^8^ Our genetic analyses provide independent, orthogonal evidence that converges on this established model.

All previously reported associations in European cohorts were replicated, and we identified 3 novel loci. The loci harboring *GATA5* and *TNFRSF10A* that were previously identified in Asian cohorts confirms that at least a subset of CSC genetic architecture is shared across ancestries. Between-study heterogeneity was low across all 10 loci, supporting robust cross-cohort replicability and accurate phenotyping in these cohorts. Genetically defined risk for CSC was also detectable as anatomical change on OCT even without a formal diagnosis of CSC. This indicates that aggregate genetic burden translates into detectable structural changes on a continuum that may be used for future studies. Of note, this is not a clinically deployable score, and we do not claim utility for individual risk stratification at present.

With integrative prioritization, we identified 10 putative causal genes (*CFH*, *CD46*, *AXL*, *PREX1*, *TGFB1*, *TNFRSF10A*, *PTPRB*, *GATA5, CASP10,* and *NOTCH4*), each supported by at least 2 independent lines of genetic evidence (eFigure 4C in Supplement 1). Among these putative causal genes, mendelian randomization and colocalization analyses pointed to 2 interesting candidates: *TGFB1* and *TNFRSF10A.* TGFB1 is a master regulator of extracellular matrix deposition and fibroblast activation,^34^ and pathological TGFB1 signaling has been implicated in scleral thickness in mammalian myopia models.^35^ We hypothesize that reduced *TGFB1* expression could alter scleral extracellular matrix composition and thereby predispose to the scleral and choroidal changes, although this mechanism has not been established experimentally. How genetically reduced TGFB1 signaling relates to the increased scleral thickness observed in CSC remains unresolved and will require direct functional and histological study. TNFRSF10A encodes TRAIL receptor 1, which is expressed on endothelial cells and has established roles in regulating endothelial apoptosis and vascular permeability.^36^ While further studies are needed to better articulate the role of TGFB1 and TNFRSF10A in CSC, circulating levels of both may contribute to CSC risk and are theoretically targetable.

In keeping with clinical observation, vascular endothelial cells were the predominant cellular carrier of CSC genetic risk across all 3 atlases. *CFH* and *CD46*, both of which are regulators of the alternative complement pathway expressed on choroidal endothelial surfaces,^38^ are in loci with the strongest overall associations. Notably, while *CFH* variants are also among the strongest risk factors for AMD, the direction of effect is opposite across the 2 diseases, suggesting that CSC and AMD represent distinct pathophysiological responses to complement dysregulation. As noted, *TNFRSF10A* has established roles in vascular biology, and *PTPRB*, a receptor tyrosine phosphatase that regulates Tie-2 signaling, was again shown to harbor a rare variant associated with CSC. This association was recently shown to co-associate with varicose veins,^16^ and that prompted a clinical case series of faricimab in patients with CSC.^40^ It is important to note that the data here contain the data from the Rämö et al single-variant study^17^ (with MGBB and an updated All of Us cohort added). The association became stronger with the addition of these data.

### Limitations

This study has limitations. First, our meta-analysis was composed of individuals of European ancestry, which may limit the generalizability of our findings to other populations. Additionally, our enrichment analysis of cell types and anatomic regions are inherently subject to the dissection and labeling limitation of the cell atlases analyzed. Few atlases that have a high-resolution, layer-specific characterization of the posterior segment. This limitation motivated our inclusion of comprehensive ocular atlases such as Tabula Sapiens. Moreover, our investigation of the individual contributions of arterial, venous, or choriocapillaris vasculature remains limited because all endothelial cells were grouped together. Our inferences regarding scleral and choroidal involvement are based on genetic and transcriptomic data, and we did not perform direct scleral or choroidal measurements; therefore, the tissue-level and cell-type enrichments reflect statistical association rather than proven causation. Case ascertainment also differed across cohorts, which may introduce phenotypic heterogeneity. Finally, the available sample was underpowered for sex-specific GWAS or sex × genotype interaction analyses; given the strong male predominance of CSC, investigation in larger cohorts is warranted.

## Conclusions

This study provides an updated comprehensive genetic characterization of CSC and provides insights into disease pathogenesis. Continued work to understand the genetics of CSC subphenotypes, the interaction of genetics with known risk factors like steroid exposure and the role of yet unanalyzed variation including exome-sequencing data are ongoing.

## Notes

Supplement 1. **eMethods 1.** Study cohorts **eMethods 2.** FinnGen ethics statement, materials and methods **eMethods 3.** Phenotype ascertainment **eMethods 4.** Quality control of genomic data **eMethods 5.** Sensitivity analysis excluding the Dutch chronic CSC cohor **eMethods 6.** Gene-based association test **eMethods 7.** Gene prioritization with Polygenic Priority Score (PoPS) **eMethods 8.** Colocalization analysis **eMethods 9.** Mendelian randomization (MR) **eMethods 10.** Cell-type enrichment across single-cell RNA-seq datasets (scDRS) **eMethods 11.** Genetic risk score and OCT review in UK Biobank **eMethods 12.** Effect Size Comparison Between CSC and AMD **eFigure 1.** Quantile–quantile plots of the individual cohort genome-wide association studies **eFigure 2.** Quantile–quantile plot of the CSC meta-analysis **eFigure 3.** Manhattan plot of the sensitivity meta-analysis excluding the Dutch cohort **eFigure 4.** Convergent genetic, proteomic and causal evidence prioritizing candidate genes **eFigure 5.** UMAP plots of CSC genetic risk across cell populations **eFigure 6.** Effect sizes of risk variants for CSC against AMD
eReferences

Supplement 2. **eTable 1.** Central serous chorioretinopathy studies and subcohorts **eTable 2.** Characteristics of the central serous chorioretinopathy patients and controls in newly analyzed biobank-based studies included in the meta-analysis **eTable 3.** Summary statistics for the 10 lead variants from the CSC genome-wide association meta-analysis **eTable 4.** Sensitivity analysis of the 10 lead variants: primary five-cohort meta-analysis compared with a meta-analysis excluding the Dutch chronic CSC cohort **eTable 5.** Gene-based association results (MAGMA) **eTable 6.** Gene prioritization results (PoPS) **eTable 7.** Bayesian colocalization results with serum protein quantitative trait loci **eTable 8.** Multiple-causal-variant colocalization (coloc.susie) results for the tested cis-pQTL regions **eTable 9.** Two-sample Mendelian randomization results **eTable 10.** Single-cell disease relevance score (scDRS) results

Supplement 3. Nonauthor collaborators: FinnGen Group

Supplement 4. Nonauthor collaborators: VA Million Veteran Program

Supplement 5. Data sharing statement
