# Supplementary material for: Genome-Wide Association Study Meta-Analysis of Central Serous Chorioretinopathy
Source: JAMA Ophthalmol. 2026 Nov 5:e264427. Online ahead of print. doi: 10.1001/jamaophthalmol.2026.4427 (PMC13741192; doi:10.1001/jamaophthalmol.2026.4427)
Supplement: Supplementary file 1 [file jamaophthalmol-e264427-s001.pdf]

Supplemental Online Content

Chen L, Kim SHF, Truong B, et al; VA Million Veteran Program; FinnGen Study. Genome-wide association study meta-analysis of central serous chorioretinopathy. *JAMA Ophthalmol*. Published online October 15, 2026. doi:10.1001/jamaophthalmol.2026.4427

eMethods 1. Study cohorts
eMethods 2. FinnGen ethics statement, materials and methods
eMethods 3. Phenotype ascertainment
eMethods 4. Quality control of genomic data
eMethods 5. Sensitivity analysis excluding the Dutch chronic CSC cohor
eMethods 6. Gene-based association test..
eMethods 7. Gene prioritization with Polygenic Priority Score (PoPS)
eMethods 8. Colocalization analysis
eMethods 9. Mendelian randomization (MR)
eMethods 10. Cell-type enrichment across single-cell RNA-seq datasets (scDRS)
eMethods 11. Genetic risk score and OCT review in UK Biobank
eMethods 12. Effect Size Comparison Between CSC and AMD
eFigure 1. Quantile–quantile plots of the individual cohort genome-wide association studies
eFigure 2. Quantile–quantile plot of the CSC meta-analysis
eFigure 3. Manhattan plot of the sensitivity meta-analysis excluding the Dutch cohort
eFigure 4. Convergent genetic, proteomic and causal evidence prioritizing candidate genes
eFigure 5. UMAP plots of CSC genetic risk across cell populations
eFigure 6. Effect sizes of risk variants for CSC against AMD
eReferences

This supplemental material has been provided by the authors to give readers additional information about their work.

© 2026 Chen L et al. *JAMA Ophthalmology*.

eMethods 1. Study cohorts

The MGBB is a biorepository of over 200,000 consented patient samples at Mass General Brigham (parent organization of Massachusetts General Hospital and Brigham and Women's Hospital, Boston, USA)[1]. It was established in 2009 and enrollment is currently ongoing. In this study, we included 53,846 genotyped participants. Samples were genotyped using 3 versions of the biobank single nucleotide polymorphism (SNP) array offered by Illumina, which is designed to capture the diversity of genetic backgrounds across the globe. Samples were imputed to TOPMed r2 reference panel using the Michigan Imputation Server[2]. Phenotypes were based on electronic health record data. EUR ancestry was assigned using a random forest classifier trained on principal components from the HGDP and 1000 Genomes reference panels, retaining individuals with a posterior probability > 0.5 for European ancestry.

FinnGen is a public-private partnership research project that combines genotype data from newly collected and legacy samples administered by Finnish biobanks (https://www.finngen.fi/en) to provide novel insight into human diseases. This study included genotype data from 486,484 individuals from FinnGen Data Freeze 12. The data were linked by unique national personal identification numbers to the national hospital discharge registry (available from 1968) and the specialist outpatient registry (1998–). EUR individuals were determined by first removing non-European outliers via principal component analysis (PCA) with 1000 Genomes, then retaining only individuals with ≥95% probability of belonging to the Finnish genetic cluster based on Mahalanobis distance.

The MVP is a population-scale biobank within the Department of Veterans Affairs. Voluntary enrollment of veterans receiving care in the Veterans Affairs (VA) began in 2011. All samples were scanned on the MVP 1.0 Axiom array (ThermoFisher). Samples were imputed to the TOPMed reference panel (version r2; N = 97,256 samples) using Minimac4 (v1.0.0). Phenotypes were based on electronic health record data with follow-up through the end of 2022. This study included genotype data from 273,904 individuals from MVP. Ancestries were inferred by integrating self-reported race/ethnicity with genetic PCA through the Harmonizing Genetic Ancestry and Self-identified Race/Ethnicity (HARE) algorithm's support vector machine[3].

The AoU Research Program opened for enrollment in May 2018 and plans to enroll at least 1 million persons in the United States, collecting electronic health record data and biospecimens to advance the prevention and treatment of diseases. In the current project, we used genetic data from 228,707 short-read whole genome-sequencing samples in the v8 release and excluded participants without linked electronic health record data. AoU assigns ancestry using a Random Forest classifier trained on HGDP[4] and 1000 Genomes reference labels across 16 principal components (PCs), requiring ≥90% predicted probability for EUR classification.

The Dutch chronic-CSC (cCSC) cohort included 521 European patients recruited from the outpatient clinics of the Radboud University Medical Center (the Netherlands), University Hospital of Cologne (Germany), and Leiden University Medical Center (the Netherlands). Controls included 3,577 participants in the Nijmegen Biomedical Study. The patients with cCSC had subretinal fluid in at least one eye, RPE irregularities with characteristic leakage on fluorescein angiography and corresponding hyperfluorescence on indocyanine green angiography. Genotyping was performed with OmniExpress-12 or OmniExpress-24 chips, and data were imputed with the Haplotype Reference Consortium (release 1.1.2016).

UKB is a deeply phenotyped and genotyped prospective population-level cohort, which recruited approximately 500,000 participants aged 40–69 in the United Kingdom (UK) between 2006–2010 (Sudlow *et al.*, 2015). UKB participants were genotyped on the Applied Biosystems UK BiLEVE Axiom Array and the Applied Biosystems UKB Axiom Array (Affymetrix, US). This research has been conducted using the UK Biobank Resource under Application Number 50211.

eMethods 2. FinnGen ethics statement, materials and methods

Study subjects in FinnGen provided informed consent for biobank research, based on the Finnish Biobank Act. Alternatively, separate research cohorts, collected prior the Finnish Biobank Act came into effect (in September 2013) and start of FinnGen (August 2017), were collected based on study-specific consents and later transferred to the Finnish biobanks after approval by Fimea (Finnish Medicines Agency), the National Supervisory Authority for Welfare and Health. Recruitment protocols followed the biobank protocols approved by Fimea. The Coordinating Ethics Committee of the Hospital District of Helsinki and Uusimaa (HUS) statement number for the FinnGen study is Nr HUS/990/2017.

© 2026 Chen L et al. *JAMA Ophthalmology*.

The FinnGen study is approved by Finnish Institute for Health and Welfare (permit numbers: THL/2031/6.02.00/2017, THL/1101/5.05.00/2017, THL/341/6.02.00/2018, THL/2222/6.02.00/2018, THL/283/6.02.00/2019, THL/1721/5.05.00/2019 and THL/1524/5.05.00/2020), Digital and population data service agency (permit numbers: VRK43431/2017-3, VRK/6909/2018-3, VRK/4415/2019-3), the Social Insurance Institution (permit numbers: KELA 58/522/2017, KELA 131/522/2018, KELA 70/522/2019, KELA 98/522/2019, KELA 134/522/2019, KELA 138/522/2019, KELA 2/522/2020, KELA 16/522/2020), Findata permit numbers THL/2364/14.02/2020, THL/4055/14.06.00/2020, THL/3433/14.06.00/2020, THL/4432/14.06/2020, THL/5189/14.06/2020, THL/5894/14.06.00/2020, THL/6619/14.06.00/2020, THL/209/14.06.00/2021, THL/688/14.06.00/2021, THL/1284/14.06.00/2021, THL/1965/14.06.00/2021, THL/5546/14.02.00/2020, THL/2658/14.06.00/2021, THL/4235/14.06.00/2021, Statistics Finland (permit numbers: TK-53-1041-17 and TK/143/07.03.00/2020 (earlier TK-53-90-20) TK/1735/07.03.00/2021, TK/3112/07.03.00/2021) and Finnish Registry for Kidney Diseases permission/extract from the meeting minutes on 4th July 2019.
The Biobank Access Decisions for FinnGen samples and data utilized in FinnGen Data Freeze 12 include: THL Biobank BB2017_55, BB2017_111, BB2018_19, BB_2018_34, BB_2018_67, BB2018_71, BB2019_7, BB2019_8, BB2019_26, BB2020_1, BB2021_65, Finnish Red Cross Blood Service Biobank 7.12.2017, Helsinki Biobank HUS/359/2017, HUS/248/2020, HUS/430/2021 §28, §29, HUS/150/2022 §12, §13, §14, §15, §16, §17, §18, §23, §58, §59, HUS/128/2023 §18, Auria Biobank AB17-5154 and amendment #1 (August 17 2020) and amendments BB_2021-0140, BB_2021-0156 (August 26 2021, Feb 2 2022), BB_2021-0169, BB_2021-0179, BB_2021-0161, AB20-5926 and amendment #1 (April 23 2020) and it´s modifications (Sep 22 2021), BB_2022-0262, BB_2022-0256, Biobank Borealis of Northern Finland_2017_1013, 2021_5010, 2021_5010 Amendment, 2021_5018, 2021_5018 Amendment, 2021_5015, 2021_5015 Amendment, 2021_5015 Amendment_2, 2021_5023, 2021_5023 Amendment, 2021_5023 Amendment_2, 2021_5017, 2021_5017 Amendment, 2022_6001, 2022_6001 Amendment, 2022_6006 Amendment, 2022_6006 Amendment, 2022_6006 Amendment_2, BB22-0067, 2022_0262, 2022_0262 Amendment, Biobank of Eastern Finland 1186/2018 and amendment 22§/2020, 53§/2021, 13§/2022, 14§/2022, 15§/2022, 27§/2022, 28§/2022, 29§/2022, 33§/2022, 35§/2022, 36§/2022, 37§/2022, 39§/2022, 7§/2023, 32§/2023, 33§/2023, 34§/2023, 35§/2023, 36§/2023, 37§/2023, 38§/2023, 39§/2023, 40§/2023, 41§/2023, Finnish Clinical Biobank Tampere MH0004 and amendments (21.02.2020 & 06.10.2020), BB2021-0140 8§/2021, 9§/2021, §9/2022, §10/2022, §12/2022, 13§/2022, §20/2022, §21/2022, §22/2022, §23/2022, 28§/2022, 29§/2022, 30§/2022, 31§/2022, 32§/2022, 38§/2022, 40§/2022, 42§/2022, 1§/2023, Central Finland Biobank 1-2017, BB_2021-0161, BB_2021-0169, BB_2021-0179, BB_2021-0170, BB_2022-0256, BB_2022-0262, BB22-0067, Decision allowing to continue data processing until 31st Aug 2024 for projects: BB_2021-0179, BB22-0067,BB_2022-0262, BB_2021-0170, BB_2021-0164, BB_2021-0161, and BB_2021-0169, and Terveystalo Biobank STB 2018001 and amendment 25th Aug 2020, Finnish Hematological Registry and Clinical Biobank decision 18th June 2021, Arctic biobank P0844: ARC_2021_1001.

eMethods 3. Phenotype ascertainment

In the MGBB cohort, patients with CSC were initially identified based on at least one instance of the ICD-10-CM code H35.71* or ICD-9-CM code 362.41. CSC diagnoses were subsequently validated through manual review of OCTs (and fluorescein angiography or indocyanine green angiography when available) by a retina specialist, and only those consistent with CSC were used as cases. Participants with AMD (ICD-10: H35.1, H35.2*, H35.3*; ICD-9: 362.5, 362.51, 362.52) were excluded.
In the All of Us cohort, patients with CSC were identified based on at least one instance of the ICD-10-CM code H35.71* or ICD−9-CM code 362.41, and all participants with age-related macular degeneration (AMD, ICD-10: H35.1*, H35.2*, H35.3*; ICD-9: 362.5, 362.51, 362.52) were excluded.
Case definitions for FinnGen, MVP, and the European chronic CSC cohort have been described previously (Rämö, Gorman, et al., 2025) and are summarized in eTable 1. Participant demographics, sex distribution, age at end of follow-up, and prevalence of pre-specified ocular comorbidities in the two newly analyzed biobank cohorts are provided in eTable 2.

eMethods 4. Quality control of genomic data

The All of Us short-read whole-genome sequencing data were pre-processed and quality-controlled centrally by the AoU Research Program, as described in the AoU Genomic Research Data Quality Report (https://support.researchallofus.org/hc/en-us/articles/29390274413716). Briefly, sample-level QC filtered on array–WGS fingerprint concordance (log-likelihood ratio > −3), sex concordance, cross-individual contamination rate (<3%), and sequencing coverage (mean coverage >30× with ≥90% of the

© 2026 Chen L et al. *JAMA Ophthalmology*.

genome covered at ≥20×), with additional removal of outliers based on per-sample variant metrics. Variant-level QC of the joint callset enforced genotype-level thresholds (genotype quality [GQ] ≥ 20, depth of coverage [DP] ≥ 10, and allele balance ≥ 0.2 at heterozygous calls), filters on excess heterozygosity (excessHet < 54.60), variant quality scores (QUAL < 60 for SNPs and < 69 for indels), excess alternate allele filters, and the AoU variant extract-train-score (VETS) filter; variants flagged by AoU were removed from downstream analyses. On top of the centralized AoU QC, we excluded any samples flagged as problematic by AoU and further filtered variants at a call rate <98%, Hardy–Weinberg equilibrium *P* value in controls $< 1\times10^{-8}$, and minor allele frequency (MAF) <1%. Principal components were computed within individuals of European ancestry using PLINK v1.9, and association testing was adjusted for sex, age, age^2, and the first ten principal components to account for residual population structure.

For the MGBB cohort QC, we excluded variants with minor allele frequency (MAF) <5%, missingness >5%, evidence of genotyping batch bias ($P < 5\times10^{-5}$), deviation from Hardy–Weinberg equilibrium at $P < 1\times10^{-10}$, and palindromic single nucleotide variants (A/T and C/G). At the sample level, we excluded individuals whose self-reported sex did not match their genetically inferred sex and individuals with an elevated heterozygosity ratio. Cleaned datasets were phased with SHAPEIT4 and imputed to the TOPMed r2 reference panel via the Michigan Imputation Server, and per-array imputed datasets were union-merged. Following imputation, we retained variants with imputation quality Rsq > 0.6 and post-imputation missingness < 5%. Principal components and continental genetic ancestry probabilities were derived by first intersecting common (MAF > 5%, genotyping rate > 95%), independent ($R^2 < 0.1$) variants between the HGDP/1000 Genomes reference panel and MGBB. PCs were calculated within HGDP/1000G samples and MGBB participants were projected into this PC space. A random forest classifier trained on the reference PCs and continental ancestry labels was then applied to MGBB, and individuals with a posterior probability > 0.5 for European ancestry were retained.

For the MVP cohort, genotyping array design, genotype calling, and the unified centralized quality-control pipeline applied to the MVP 1.0 Axiom array have been described in detail previously[5]. Briefly, the unified QC pipeline applied to an initial release of 485,856 genotyped MVP participants retained 459,777 unique individuals and 668,418 high-quality markers, with high call performance across both common and rare variants.

The FinnGen genotype imputation protocol is available at: https://dx.doi.org/10.17504/protocols.io.xbgfijw. Estonian Biobank samples were genotyped and PLINK format files were created using Illumina GenomeStudio v2.0.4. Individuals were excluded from the analysis if their call-rate was < 95% or if sex defined based on heterozygosity of X chromosome did not match sex in phenotype data. Before imputation, variants were filtered by call-rate < 95%, Hardy–Weinberg equilibrium *P* value $< 1\times10^{-4}$ (autosomal variants only), and minor allele frequency < 1%. Variant positions were in build 37 and all variants were changed to be from TOP strand using GSAMD-24v1-0_20011747_A1-b37.strand.RefAlt.zip files from https://www.well.ox.ac.uk/~wrayner/strand/ webpage. Pre-phasing was performed using Eagle v2.3 software. The number of conditioning haplotypes Eagle2 uses when phasing each sample was set to: --Kpbwt=20000 and imputation was performed using Beagle v.28Sep18.79 with effective population size ne=20,000.

Quality control of the Dutch cCSC cohort was performed in PLINK v1.90 separately within each batch and again after merging. Within each batch, samples were excluded if their genotyping call rate was <97% or if reported sex disagreed with genotype-inferred sex (71 controls and 7 cases removed), and variants were excluded if their call rate was <98%, if they deviated from Hardy–Weinberg equilibrium at $P < 1\times10^{-6}$, or if their minor allele frequency was <1%. The most recent case batch was lifted over from GRCh38 to GRCh37 to match the other batches using the UCSC LiftOver tool. Variants common to all batches were retained, the batches were merged, and strand inconsistencies were resolved by iterative flipping using the PLINK flipscan utility until concordance was reached across all datasets. Variants with a post-merge call rate <98% were then removed, leaving 589,487 autosomal and 13,282 X-chromosomal variants for downstream analysis. Population stratification was assessed by merging the cohort with HapMap3, pruning at a 50 kb window / step 5 / VIF 2, and principal-component analysis in PLINK; 2 controls and 13 cases identified as non-European outliers were excluded. Cryptic relatedness was evaluated with KING v2.0, and 4 controls and 5 cases exceeding a kinship coefficient of 0.0884 (duplicates and first- or second-degree relatives) were removed. After QC, 521 cCSC cases and 3,577 NBS controls remained. Autosomal variants were phased with Eagle v2.3 and X-chromosomal variants with SHAPEIT v2.r790, and the merged dataset was imputed to the Haplotype Reference Consortium

© 2026 Chen L et al. *JAMA Ophthalmology*.

reference panel (release 1.1.2016) using the Michigan Imputation Server. SNPs were filtered on an imputation quality score of $R2 > 0.3$ for common variants (MAF >5%) and a $R2 > 0.8$ for low frequency variants (MAF <5%).

eMethods 5. Sensitivity analysis excluding the Dutch chronic CSC cohort

Case ascertainment in the Dutch cohort differed from that in the other four cohorts. Patients were recruited clinically and met a previously published definition of chronic CSC, whereas cases in FinnGen, All of Us, the Mass General Brigham Biobank and the Million Veteran Program were identified from diagnosis codes. To assess whether this difference influenced the meta-analysis, we repeated the fixed-effects inverse-variance-weighted meta-analysis in GWAMA[6] v2.2.2 using only the four code-based cohorts (2,063 cases), applying the same variant inclusion criterion of presence in at least 2 cohorts. Effect estimates were expressed with respect to the effect allele of the primary analysis; alignment was confirmed for all 10 lead variants by agreement of effect-allele frequency (maximum absolute difference 0.008) and concordance of the sign of beta, and no estimate required sign reversal.

All 10 lead variants retained the same direction of effect, with per-allele log-odds ratios between 0.82 and 1.07 times the corresponding primary estimates. Six of the 10 lead variants, and 7 of the 10 loci, retained $P < 5 \times 10^{-8}$ (eTable 4 and eFigure 3). The *GATA5* locus remained below the genome-wide threshold through a variant 4.2 kb from the primary lead ($P = 1.33 \times 10^{-8}$) rather than through the lead variant itself ($P = 7.44 \times 10^{-8}$). The loci nearest *PREX1*, *TNFRSF10A* and *LINC00551* did not reach the genome-wide threshold after exclusion ($P = 1.01 \times 10^{-5}$, 1.19×10^{-5} and 4.42×10^{-6}, respectively). These were the three weakest associations in the primary analysis, and their attenuation is consistent with the 20% reduction in case count rather than with any change in the direction or magnitude of effect.

Between-study heterogeneity was lower after exclusion at 5 of the 10 loci, essentially unchanged at 4, and slightly higher at the *PTPRB* locus (I^2 0.22 to 0.25); mean I^2 fell from 0.17 to 0.10. Taken together, these results indicate that the primary meta-analysis is robust to the difference in case ascertainment in the Dutch cohort, with the expected loss of power at the weakest loci when 20% of cases are removed.

eMethods 6. Gene-based association test

We performed gene-based tests on the meta-analysis summary statistic Z-scores using MAGMA v1.10[7]. SNPs were mapped to protein-coding genes following the Ensembl gene model[8]. Gene-wise *P* values were computed under the SNP-wise mean model using 1000 Genomes Phase 3 EUR as LD reference. MAF was set to a threshold of ≥0.001, and a 10kb window around gene start and stop. 6,535,749 SNPs (64.25%) were mapped to at least one gene, and SNPs were mapped to 17,832 genes. Gene-based *P* values were calculated using the SNP-wise Mean model. Multiple testing was controlled by Bonferroni for gene-based tests ($\alpha = .05/17{,}832$).

eMethods 7. Gene prioritization with Polygenic Priority Score (PoPS)

We prioritized putative effector genes using the PoPS[9] framework applied to MAGMA gene-level association statistics. PoPS was run with the full feature panel distributed by the original PoPS study. Following the recommended PoPS workflow, we trained a ridge regression model with internal cross-validation on the genome-wide set of genes and generated out-of-fold PoPS scores for each gene to avoid overfitting. We retained only positively scored genes when ranking within loci. For locus-level gene nomination, we selected the top 2 PoPS-scoring genes within each locus to create a locus-specific candidate list.

eMethods 8. Colocalization analysis

We performed Bayesian colocalization analysis using the *coloc*[10] package (version 5.2.3) to assess whether serum protein quantitative trait loci (pQTLs) from the UKB[11] and genetic variants associated with CSC risk share the same underlying causal variants. pQTL summary statistics were downloaded from Synapse (https://www.synapse.org/Synapse:syn51364943). We specified prior probabilities ($p_1 = 1\times10^{-4}$, $p_2 = 1\times10^{-4}$, and $p_{12} = 1\times10^{-5}$) consistent with the typical genetic architecture of GWAS and molecular QTL studies[12]. The outputs of coloc are the probabilities of five mutually exclusive hypotheses for a genomic region (H_0: No association with either trait; H_1: Association with trait 1 only; H_2: Association with trait 2 only; H_3: Both traits associated, but with different causal variants; H_4: Both traits

© 2026 Chen L et al. *JAMA Ophthalmology*.

associated with the same shared causal variant). We defined strong colocalization evidence as PP4/PP3 > 3 and PP4 ≥ 0.9. The PP4 threshold follows established practice[12], while the additional PP4/PP3 ratio requirement reduces false-positive signals driven by residual uncertainty rather than clear differentiation from H_3[13].

eMethods 9. Mendelian randomization (MR)

We performed two-sample MR[14] to proteins with strong colocalization evidence, using pQTL from the UKB proteomics GWAS as exposures and our CSC meta-analysis as the outcome. For each protein, we restricted instruments to cis variants within the gene±100 kb window and required association evidence at $P \leq .01$ in the pQTL summary statistics. To obtain approximately independent instruments, we LD-clumped the cis-eligible variants with PLINK 2.0[15] against a 1000 Genomes European (hg38) reference panel ($r^2 \leq 0.10$, 250 kb window, p1 = 5×10^{-8}, p2 = 0.01). For MR estimation, we used inverse-variance weighted (IVW), simple median, and weighted median estimators. A protein was carried forward only when at least 2 of the 3 estimators yielded estimates below the Bonferroni-corrected threshold with concordant direction. We assessed directional horizontal pleiotropy using the MR-Egger intercept test. The MR-Egger slope was reported as a sensitivity estimate when ≥3 instruments are available. An MR-Egger intercept with $P > .05$ was interpreted as no evidence of directional horizontal pleiotropy. Multiple testing was controlled by Bonferroni (α = .05) across the number of colocalized proteins carried forward.

eMethods 10. Cell-type enrichment across single-cell RNA-seq datasets (scDRS)

We assessed cellular enrichment of CSC genetic signals using the Single-Cell Disease Relevance Score (scDRS) method[16]. The prioritized set of genes from PoPS, colocalization and MR were used as input. Three independent human datasets were used: a retinal atlas[17], an ocular posterior segment atlas[18], and the multi-organ Tabula Sapiens resource[19,20]. The analyses reported here used the 2022 Tabula Sapiens release, which is an expanded release spanning 28 tissues.

For scDRS scoring, we used the full score mode with b = 5,000 matched control gene sets per trait, mean–variance control matching (--ctrl-match-opt mean_var), and variance-stabilized weighting (--weight-opt vs). Covariates included donor identifier and sex for all datasets; where available we additionally regressed out technical covariates (e.g., number of detected genes, total counts, and mitochondrial percentage). We required a minimum of 50 cells per group for inference. The primary inferential endpoint was the group-level association test ("assoc") implemented in scDRS. Multiple testing was controlled using Benjamini–Hochberg FDR within each dataset and grouping variable (e.g., within cell-type labels or tissue compartments).

eMethods 11. Genetic risk score and OCT review in UK Biobank

We constructed a CSC GRS using the 10 lead variants identified in the CSC meta-analysis (Table 1), weighted by meta-analysis log-odds ratios. We matched effect alleles between the meta-analysis and UKB genotypes, removed strand-ambiguous markers without clear MAF alignment and computed per-individual GRS using the *--score* function in PLINK 1.9 (Igo, Kinzy and Cooke Bailey, 2019). We restricted the analysis to unrelated European-ancestry participants with array genotype data and macular OCT scans. Among unrelated UK Biobank participants of European ancestry with array genotype data, those falling in the prespecified top and bottom 1% of the CSC genetic risk score distribution were identified (*n* = 4,449), of whom 1,558 had macular OCT imaging. Because grading required manual review by a retina specialist, a random subset of 250 of these participants was selected for review. Seven scans were of insufficient quality to interpret and were recorded as ungradable rather than scored, leaving 243 participants for analysis (top 1%, *n* = 109; bottom 1%, *n* = 134).

All scans were graded by a single attending retina specialist (EJR) masked to genetic risk group, and the analyst (LC) was likewise masked. A participant was classified as having a CSC-related abnormality when at least one of the prespecified findings was present in either eye; scans with no such finding were classified as normal. Findings were counted only when their distribution was consistent with current or previous subretinal fluid, since the individual features are not specific to CSC in isolation. Subretinal fluid and evidence of CSC were recorded as separate options during grading to give the grader flexibility, but both collapse to the same binary outcome in the analysis. Pachychoroid pigment epitheliopathy was removed from the list of scored findings because the term lacks an agreed definition; the single participant scored on this basis also had a pigment epithelial detachment and therefore remains

© 2026 Chen L et al. *JAMA Ophthalmology*.

classified as abnormal, so the counts are unchanged. Five participants (2%) were scored on the basis of pattern dystrophy-like changes. True pattern dystrophy cannot be reliably distinguished from CSC-related pigmentary change on OCT alone, and this attribution is therefore presumptive.

Participants were not excluded on the basis of other diagnoses. Age-related macular degeneration was not an exclusion criterion, but AMD-specific features, including drusen, were recorded without being counted as CSC-consistent abnormalities.

The 1% threshold was fixed in advance and no other threshold was evaluated. It was chosen for two reasons. First, complete masked grading by a retina specialist was feasible only at this scale; widening to the top and bottom 5% would require several thousand scans per tail, and a decile analysis roughly twice that. Second, at the present case count the genetic risk score is expected to discriminate poorly through the middle of its distribution, so a quintile, decile or continuous analysis would be expected to carry high false-positive and false-negative rates. This analysis is therefore a proof of concept confined to the extremes of the distribution, and we do not claim that the score is clinically useful in its present form.

eMethods 12. Effect Size Comparison Between CSC and AMD

Because the CSC GWAS is still relatively underpowered for formal genetic correlation analysis, we compared effect sizes at CSC loci identified here and previously reported AMD loci[21]**.** For each locus, the lead variant was used when available in both datasets; otherwise, a proxy variant in linkage disequilibrium was chosen. Proxy variants were precomputed using the 1000 Genomes Phase 3 EUR reference panel within ±50 kb windows around each lead variant and ranked by linkage disequilibrium (R^2), with the highest-R^2 proxy selected among those present in both datasets. Only loci for which either the lead variants or eligible proxies were available and had a *P* value below the nominal threshold ($P < .05$) in both datasets were included. Effect sizes were harmonized to a common effect allele prior to analysis. Pearson correlation coefficients and linear regression lines were computed to quantify cross-trait concordance.

© 2026 Chen L et al. *JAMA Ophthalmology*.

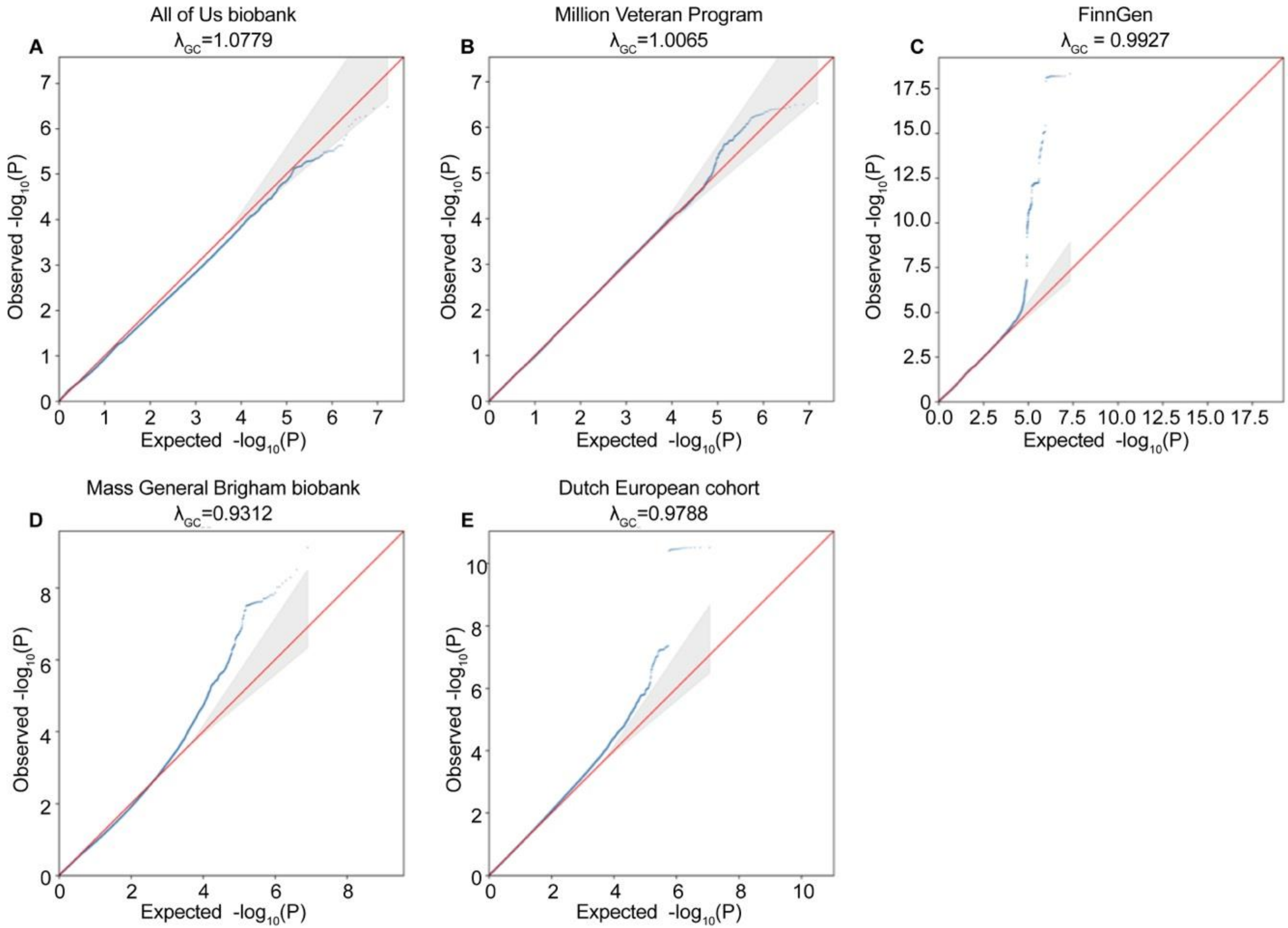

© 2026 Chen L et al. *JAMA Ophthalmology*.

eFigure 1. Quantile-Quantile (Q-Q) Plots of Individual Cohort GWAS Results. Plots display the observed versus expected $-\log_{10}(P)$ values for the five independent cohorts contributed to the meta-analysis: A, All of Us biobank; B, Million Veteran Program; C, FinnGen; D, Mass General Brigham Biobank; and E, the Dutch European cohort. The genomic inflation factor (λGC) is provided for each cohort. The red line indicates the null hypothesis of no association (slope = 1), and the shaded gray region represents the 95% confidence interval. The λGC values near 1.0 indicate adequate control for population stratification and cryptic relatedness prior to meta-analysis.

© 2026 Chen L et al. *JAMA Ophthalmology*.

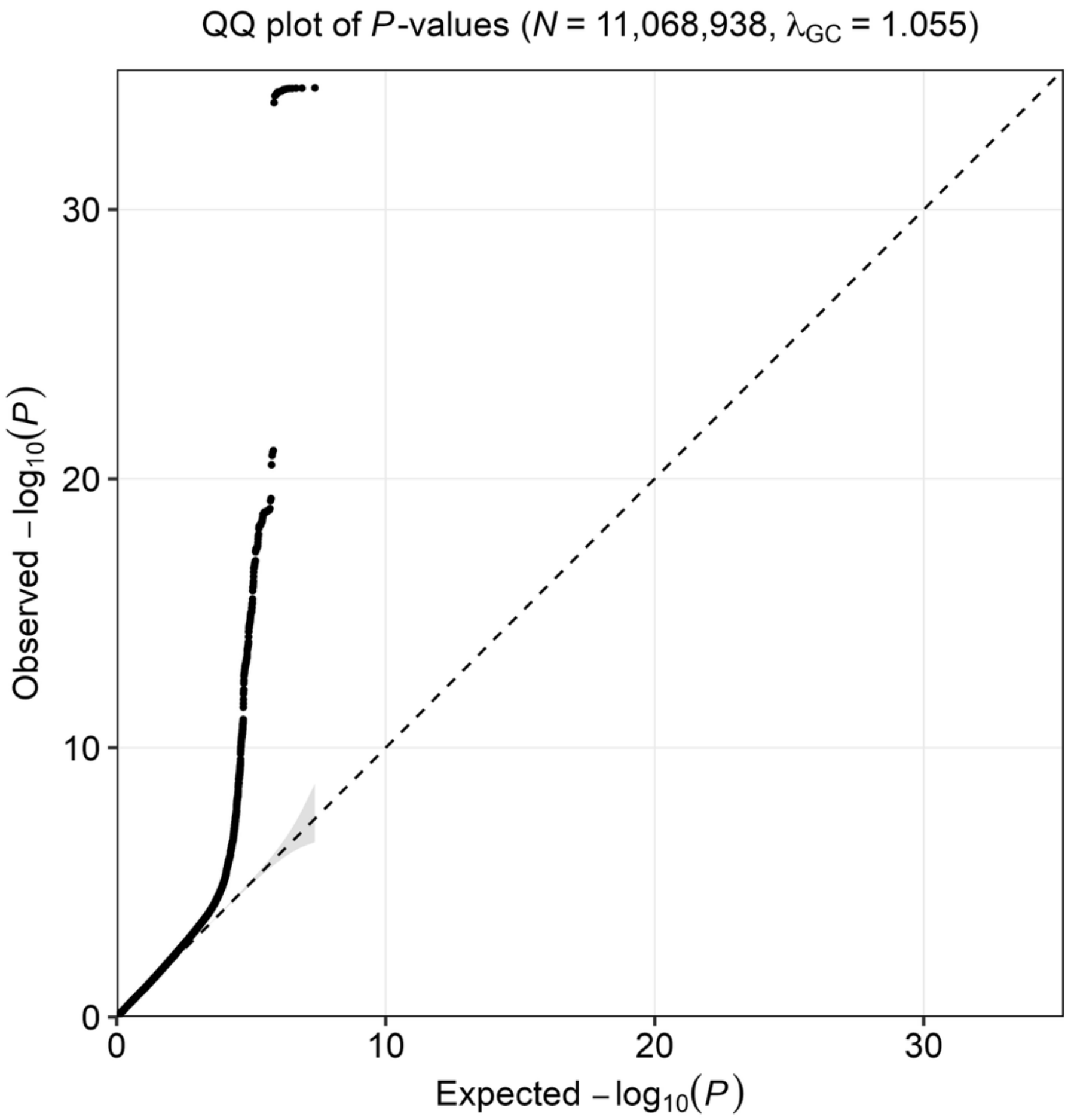

eFigure 2. Quantile-quantile (Q-Q) plot of the CSC meta-analysis.

© 2026 Chen L et al. *JAMA Ophthalmology*.

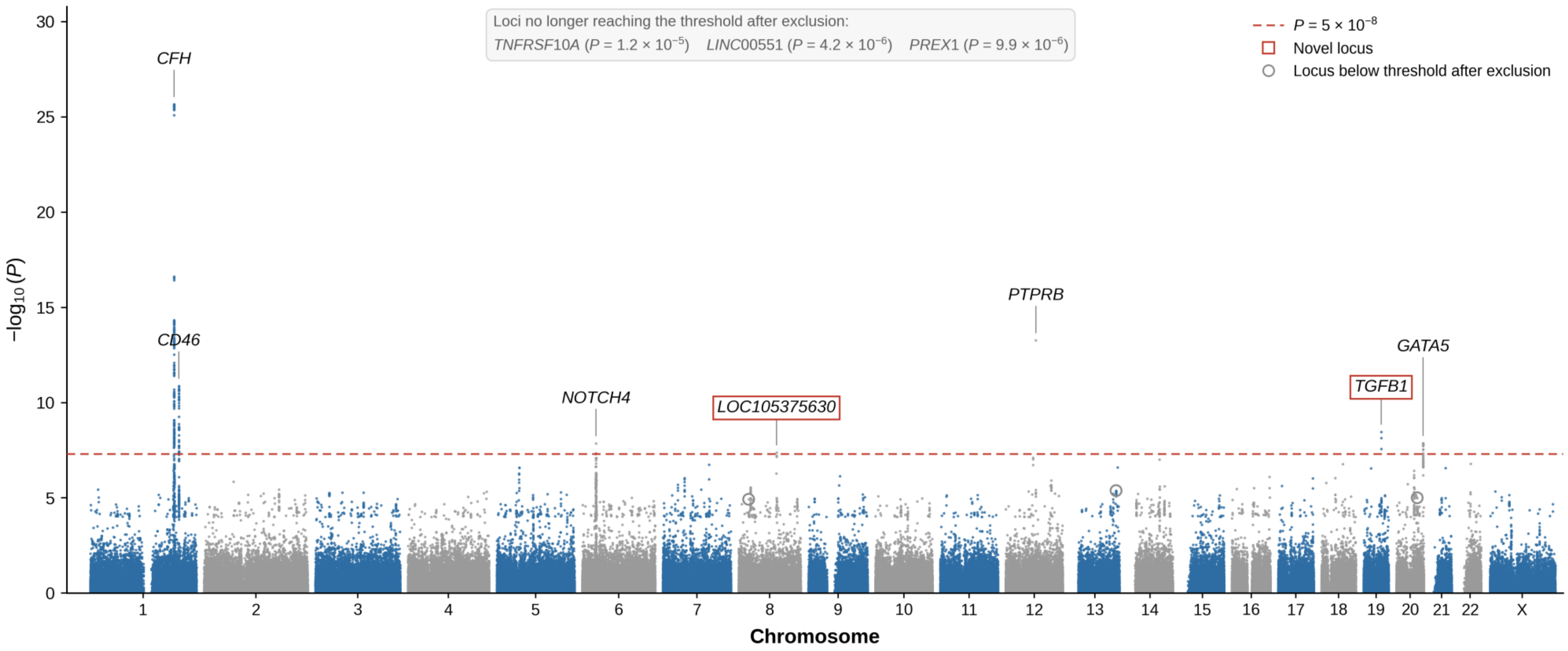

eFigure 3. Manhattan plot of the sensitivity meta-analysis excluding the Dutch chronic CSC cohort. Fixed-effects inverse-variance-weighted meta-analysis of the four cohorts in which cases were ascertained from diagnosis codes (FinnGen, All of Us, the Mass General Brigham Biobank and the Million Veteran Program; 2,063 cases), restricted to variants present in at least 2 cohorts. Each point represents a single variant and chromosomes alternate in colour. The horizontal dashed red line marks the genome-wide threshold ($P = 5 \times 10^{-8}$). The nearest gene is annotated for each locus reaching this threshold, with novel loci labelled in red boxes. Open grey circles mark the three loci that no longer reach the threshold after exclusion, whose P values are given in the panel note. Effect estimates for the 10 lead variants in both the primary and the reduced analysis are provided in eTable 4. For legibility, variants with $P > 1 \times 10^{-4}$ are plotted as a random 1-in-25 sample; all variants with $P \leq 1 \times 10^{-4}$ are shown.

© 2026 Chen L et al. *JAMA Ophthalmology*.

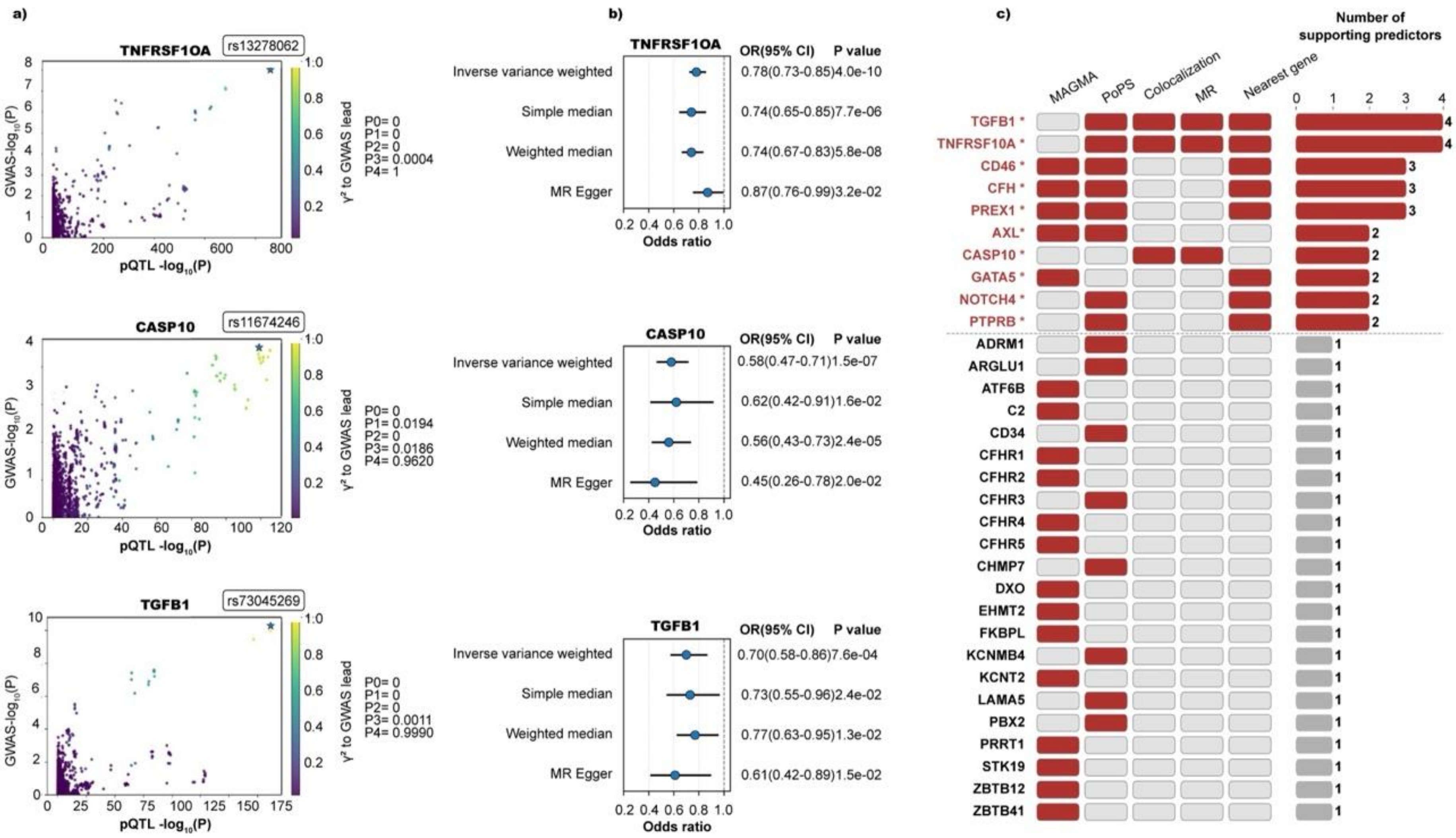

eFigure 4. Convergent genetic, proteomic, and causal evidence prioritizes candidate genes for CSC. (a) LocusCompare plots of the 3 proteins with strong colocalization (PP4 > 0.95) between the cis-pQTL and the CSC GWAS signal. x-axis shows the P value of each variant in the serum protein pQTL, and the y-axis shows the P value of each variant in the CSC meta-analysis. The right column reports the posterior probability estimates from coloc. b)

© 2026 Chen L et al. *JAMA Ophthalmology*.

Forest plot of MR estimates for the same proteins shown in (a). For each protein, points show the causal odds ratio (OR) for CSC per genetically predicted increase in protein level. Rows within each panel correspond to complementary MR estimators. The vertical dashed line marks the null (OR = 1). The two right columns report the OR (95% CI) and the P value for each method (Bonferroni-corrected threshold: P < .05/3 = .017). (c) Binary heatmap showing the mapping of candidate genes (rows) to 5 gene prioritization criteria (columns): MAGMA, PoPS, colocalization, MR, and nearest gene. Red cells indicate that a gene was identified by the corresponding method; grey cells indicate no support. Genes supported by 2 or more independent predictors (above dashed line, bold red labels with asterisks) were designated as prioritized genes. Of 33 candidate genes, ten were prioritized based on convergent evidence.

© 2026 Chen L et al. *JAMA Ophthalmology*.

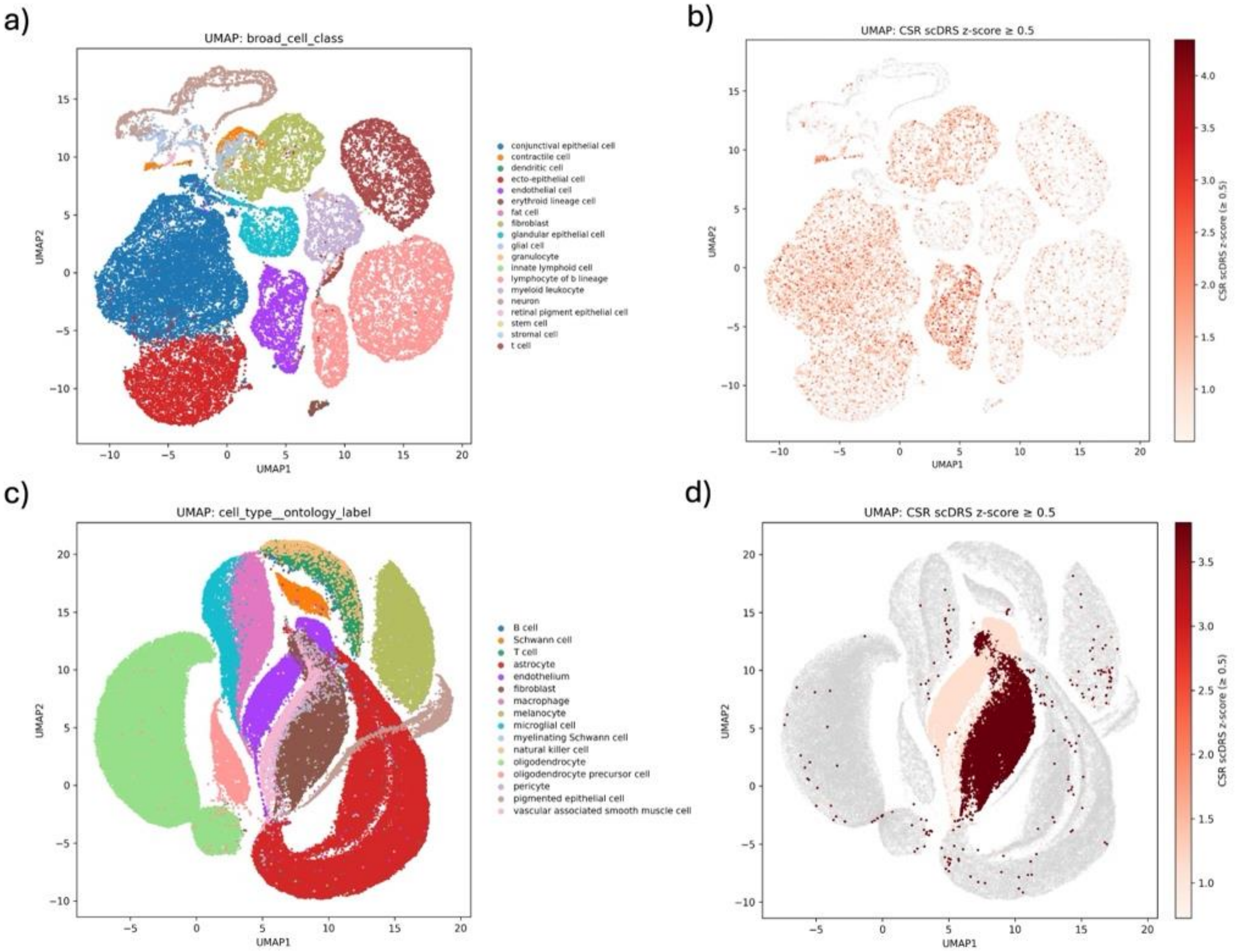

© 2026 Chen L et al. *JAMA Ophthalmology*.

eFigure 5. Uniform Manifold Approximation and Projection (UMAP) plots visualizing the enrichment of CSC genetic risk within specific cell populations. a) UMAP of the Tabula Sapiens multi-organ atlas (ocular subset) colored by annotated cell type. b) The same UMAP colored by CSC scDRS z-score. Darker red indicates a higher aggregate genetic risk score for that individual cell. Note the elevated signal in endothelial and fibroblast clusters. c) UMAP of the Ocular Posterior Segment Atlas colored by annotated cell type. d) The same UMAP colored by CSC scDRS z-score. The strongest signal (dark red) corresponds to the endothelial cell cluster (purple in panel C), indicating that CSC risk variants are preferentially active in vascular endothelium.

© 2026 Chen L et al. *JAMA Ophthalmology*.

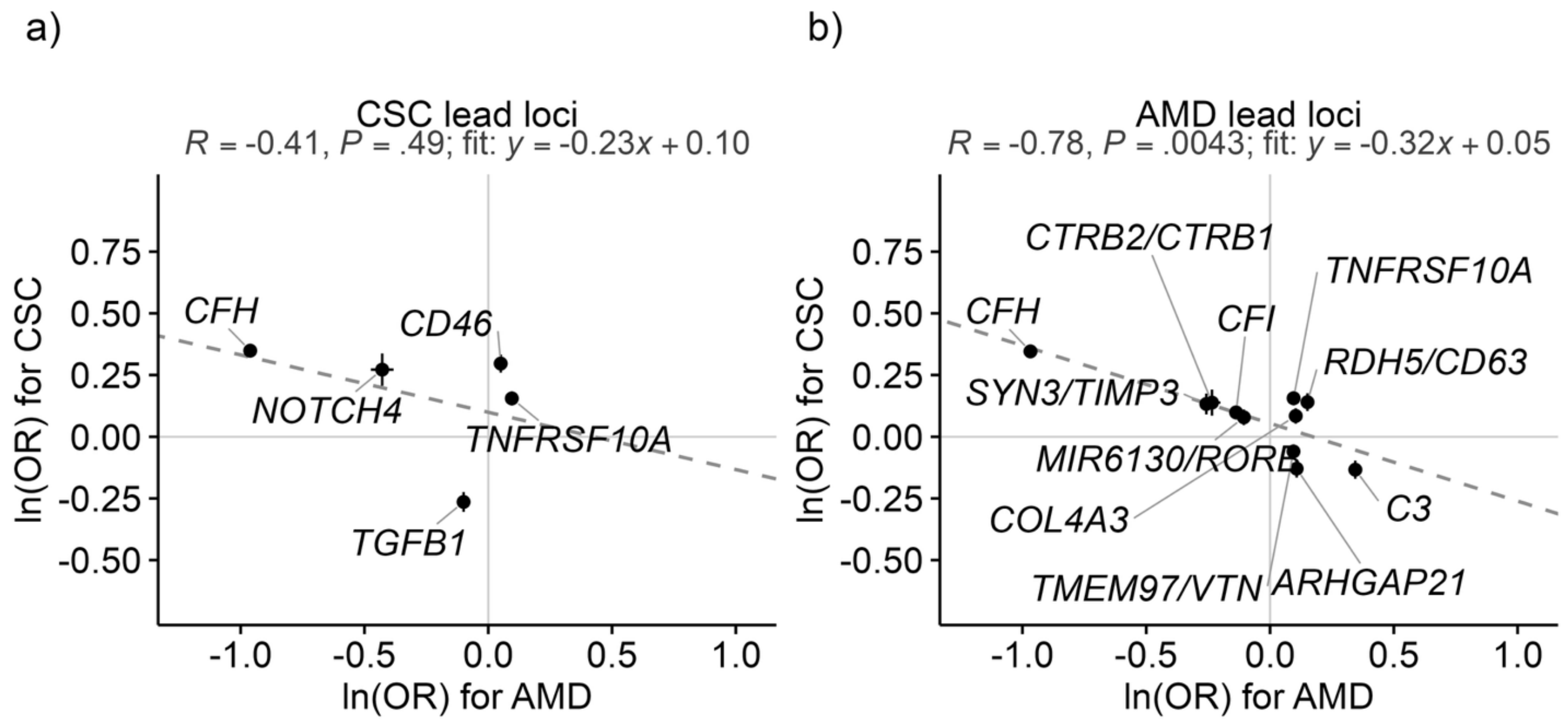

eFigure 6. Scatterplots comparing the log-odds ratios (Beta) of risk variants for CSC (y-axis) against AMD (x-axis). a) Correlation of effect sizes at the CSC risk loci reaching the genome-wide threshold in this study. A similar negative trend is observed (R = -0.41), with a less precisely estimated correlation (P = .49), likely due to the smaller number of available index variants. Error bars represent standard errors. b) Correlation of effect sizes at established AMD risk loci (Fritsche et al.). An inverse correlation (R = -0.78; P = .004) indicates that variants increasing risk for AMD are generally protective for CSC.

© 2026 Chen L et al. *JAMA Ophthalmology*.

eReferences

1. Koyama S, Wang Y, Paruchuri K, et al. Genetics and context for precision health in Greater Boston. *Nat Commun*. 2025;16(1):11661. doi:10.1038/s41467-025-66598-8

2. Das S, Forer L, Schönherr S, et al. Next-generation genotype imputation service and methods. *Nat Genet*. 2016;48(10):1284-1287. doi:10.1038/ng.3656

3. Fang H, Hui Q, Lynch J, et al. Harmonizing Genetic Ancestry and Self-identified Race/Ethnicity in Genome-wide Association Studies. *Am J Hum Genet*. 2019;105(4):763-772. doi:10.1016/j.ajhg.2019.08.012

4. Bergström A, McCarthy SA, Hui R, et al. Insights into human genetic variation and population history from 929 diverse genomes. *Science*. 2020;367(6484):eaay5012. doi:10.1126/science.aay5012

5. Hunter-Zinck H, Shi Y, Li M, et al. Genotyping Array Design and Data Quality Control in the Million Veteran Program. *Am J Hum Genet*. 2020;106(4):535-548. doi:10.1016/j.ajhg.2020.03.004

6. Mägi R, Morris AP. GWAMA: software for genome-wide association meta-analysis. *BMC Bioinformatics*. 2010;11:288. doi:10.1186/1471-2105-11-288

7. de Leeuw CA, Mooij JM, Heskes T, Posthuma D. MAGMA: generalized gene-set analysis of GWAS data. *PLoS Comput Biol*. 2015;11(4):e1004219. doi:10.1371/journal.pcbi.1004219

8. Martin FJ, Amode MR, Aneja A, et al. Ensembl 2023. *Nucleic Acids Res*. 2023;51(D1):D933-D941. doi:10.1093/nar/gkac958

9. Weeks EM, Ulirsch JC, Cheng NY, et al. Leveraging polygenic enrichments of gene features to predict genes underlying complex traits and diseases. *Nat Genet*. 2023;55(8):1267-1276. doi:10.1038/s41588-023-01443-6

10. Giambartolomei C, Vukcevic D, Schadt EE, et al. Bayesian Test for Colocalisation between Pairs of Genetic Association Studies Using Summary Statistics. Williams SM, ed. *PLoS Genet*. 2014;10(5):e1004383. doi:10.1371/journal.pgen.1004383

11. Sun BB, Chiou J, Traylor M, et al. Plasma proteomic associations with genetics and health in the UK Biobank. *Nature*. 2023;622(7982):329-338. doi:10.1038/s41586-023-06592-6

12. Wallace C. Eliciting priors and relaxing the single causal variant assumption in colocalisation analyses. *PLoS Genet*. 2020;16(4):e1008720. doi:10.1371/journal.pgen.1008720

13. Tokolyi A, Persyn E, Nath AP, et al. The contribution of genetic determinants of blood gene expression and splicing to molecular phenotypes and health outcomes. *Nat Genet*. 2025;57(3):616-625. doi:10.1038/s41588-025-02096-3

14. Hemani G, Zheng J, Elsworth B, et al. The MR-Base platform supports systematic causal inference across the human phenome. *eLife*. 2018;7:e34408. doi:10.7554/eLife.34408

15. Chang CC, Chow CC, Tellier LC, Vattikuti S, Purcell SM, Lee JJ. Second-generation PLINK: rising to the challenge of larger and richer datasets. *Gigascience*. 2015;4:7. doi:10.1186/s13742-015-0047-8

16. Zhang MJ, Hou K, Dey KK, et al. Polygenic enrichment distinguishes disease associations of individual cells in single-cell RNA-seq data. *Nat Genet*. 2022;54(10):1572-1580. doi:10.1038/s41588-022-01167-z

17. Menon M, Mohammadi S, Davila-Velderrain J, et al. Single-cell transcriptomic atlas of the human retina identifies cell types associated with age-related macular degeneration. *Nat Commun*. 2019;10(1):4902. doi:10.1038/s41467-019-12780-8

© 2026 Chen L et al. *JAMA Ophthalmology*.

18. Monavarfeshani A, Yan W, Pappas C, et al. Transcriptomic analysis of the ocular posterior segment completes a cell atlas of the human eye. *Proc Natl Acad Sci U S A*. 2023;120(34):e2306153120. doi:10.1073/pnas.2306153120

19. Tabula Sapiens Consortium*, Jones RC, Karkanias J, et al. The Tabula Sapiens: A multiple-organ, single-cell transcriptomic atlas of humans. *Science*. 2022;376(6594):eabl4896. doi:10.1126/science.abl4896

20. The Tabula Sapiens Consortium, Quake SR. Tabula Sapiens reveals transcription factor expression, senescence effects, and sex-specific features in cell types from 28 human organs and tissues. *Cell Biology*. Preprint posted online December 4, 2024. doi:10.1101/2024.12.03.626516

21. Fritsche LG, Igl W, Bailey JNC, et al. A large genome-wide association study of age-related macular degeneration highlights contributions of rare and common variants. *Nat Genet*. 2016;48(2):134-143. doi:10.1038/ng.3448

© 2026 Chen L et al. *JAMA Ophthalmology*.
