# Supplementary material for: Genome-Wide Association Study Meta-Analysis of Central Serous Chorioretinopathy
Source: JAMA Ophthalmol. 2026 Nov 5:e264427. Online ahead of print. doi: 10.1001/jamaophthalmol.2026.4427 (PMC13741192; doi:10.1001/jamaophthalmol.2026.4427)
Supplement: Supplementary file 2 [file jamaophthalmol-e264427-s003.pdf]

Supplemental Online Content: Nonauthor Collaborators
*First name, last name, and suffix (if applicable) are required and will appear in PubMed.

*Group Name(s): FinnGen Study							
***First Name and Middle Initial(s)**	***Last Name**	***Suffix (eg, Jr, III)**	Academic Degrees	Institution	Location (city, state/province, country)	Role or Contribution, eg, chair, principal investigator	Group (if more than 1 Group listed in the byline) and/or Subgroup (eg, Steering Committee)
Bridget	Riley-Gills			Abbvie	Chicago, IL, United States		FinnGen Study; Steering Committee (Pharmaceutical companies)
Howard	Jacob			Abbvie	Chicago, IL, United States		FinnGen Study; Steering Committee (Pharmaceutical companies)
Coralie	Viollet			AstraZeneca	Cambridge, United Kingdom		FinnGen Study; Steering Committee (Pharmaceutical companies)
Slavé	Petrovski			AstraZeneca	Cambridge, United Kingdom		FinnGen Study; Steering Committee (Pharmaceutical companies); Scientific Committee (Pharmaceutical companies)
Alix	Berton			Bayer AG	Leverkusen, Germany		FinnGen Study; Steering Committee (Pharmaceutical companies); Scientific Committee (Pharmaceutical companies)

Supplemental Online Content: Nonauthor Collaborators
*First name, last name, and suffix (if applicable) are required and will appear in PubMed.

***First Name and Middle Initial(s)**	***Last Name**	***Suffix (eg, Jr, III)**	Academic Degrees	Institution	Location (city, state/province, country)	Role or Contribution, eg, chair, principal investigator	Group (if more than 1 Group listed in the byline) and/or Subgroup (eg, Steering Committee)
Santha	Ramakrishnan			Bayer AG	Leverkusen, Germany		FinnGen Study; Steering Committee (Pharmaceutical companies); Scientific Committee (Pharmaceutical companies)
Ellen	Tsai			Biogen	Cambridge, MA, United States		FinnGen Study; Steering Committee (Pharmaceutical companies); Scientific Committee (Pharmaceutical companies)
Zhihao	Ding			Boehringer Ingelheim	Ingelheim am Rhein, Germany		FinnGen Study; Steering Committee (Pharmaceutical companies); Scientific Committee (Pharmaceutical companies)
Emily	Holzinger			Bristol Myers Squibb	New York, NY, United States		FinnGen Study; Steering Committee (Pharmaceutical companies)
Robert	Plenge			Bristol Myers Squibb	New York, NY, United States		FinnGen Study; Steering Committee (Pharmaceutical companies)

Supplemental Online Content: Nonauthor Collaborators
*First name, last name, and suffix (if applicable) are required and will appear in PubMed.

***First Name and Middle Initial(s)**	***Last Name**	***Suffix (eg, Jr, III)**	Academic Degrees	Institution	Location (city, state/province, country)	Role or Contribution, eg, chair, principal investigator	Group (if more than 1 Group listed in the byline) and/or Subgroup (eg, Steering Committee)
Joseph	Maranville			Bristol Myers Squibb	New York, NY, United States		FinnGen Study; Steering Committee (Pharmaceutical companies)
Mark	McCarthy			Genentech	San Francisco, CA, United States		FinnGen Study; Steering Committee (Pharmaceutical companies); Clinical Group / Task Force
Rion	Pendergrass			Genentech	San Francisco, CA, United States		FinnGen Study; Steering Committee (Pharmaceutical companies); Scientific Committee (Pharmaceutical companies); Clinical Group / Task Force
Jonathan	Davitte			GlaxoSmithKline	Collegeville, PA, United States		FinnGen Study; Steering Committee (Pharmaceutical companies); Scientific Committee (Pharmaceutical companies)
Chia-Yen	Chen			Merck	Kenilworth, NJ, United States		FinnGen Study; Steering Committee (Pharmaceutical companies)

Supplemental Online Content: Nonauthor Collaborators
*First name, last name, and suffix (if applicable) are required and will appear in PubMed.

***First Name and Middle Initial(s)**	***Last Name**	***Suffix (eg, Jr, III)**	Academic Degrees	Institution	Location (city, state/province, country)	Role or Contribution, eg, chair, principal investigator	Group (if more than 1 Group listed in the byline) and/or Subgroup (eg, Steering Committee)
Melis	Atalar Aksit			Pfizer	New York, NY, United States		FinnGen Study; Steering Committee (Pharmaceutical companies); Scientific Committee (Pharmaceutical companies)
Anna	Vlahiotis			Pfizer	New York, NY, United States		FinnGen Study; Steering Committee (Pharmaceutical companies)
Katherine	Klinger			Translational Sciences, Sanofi R&D	Framingham, MA, USA		FinnGen Study; Steering Committee (Pharmaceutical companies); Scientific Committee (Pharmaceutical companies)
Clement	Chatelain			Translational Sciences, Sanofi R&D	Framingham, MA, USA		FinnGen Study; Steering Committee (Pharmaceutical companies)
Jorg	Blankenstein			Translational Sciences, Sanofi R&D	Framingham, MA, USA		FinnGen Study; Steering Committee (Pharmaceutical companies)
Karol	Estrada			Maze Therapeutics	San Francisco, CA, United States		FinnGen Study; Steering Committee (Pharmaceutical companies)

*First name, last name, and suffix (if applicable) are required and will appear in PubMed.

***First Name and Middle Initial(s)**	***Last Name**	***Suffix (eg, Jr, III)**	Academic Degrees	Institution	Location (city, state/province, country)	Role or Contribution, eg, chair, principal investigator	Group (if more than 1 Group listed in the byline) and/or Subgroup (eg, Steering Committee)
Robert	Graham			Maze Therapeutics	San Francisco, CA, United States		FinnGen Study; Steering Committee (Pharmaceutical companies); Scientific Committee (Pharmaceutical companies)
Dawn	Waterworth			Johnson & Johnson Innovative Medicine	Spring House, PA, United States		FinnGen Study; Steering Committee (Pharmaceutical companies); Scientific Committee (Pharmaceutical companies)
Chris	O´Donnell			Novartis Institutes for BioMedical Research	Cambridge, MA, United States		FinnGen Study; Steering Committee (Pharmaceutical companies)
Nicole	Renaud			Novartis Institutes for BioMedical Research	Cambridge, MA, United States		FinnGen Study; Steering Committee (Pharmaceutical companies); Scientific Committee (Pharmaceutical companies)
Tomi P.	Mäkelä			HiLIFE, University of Helsinki	Finland		FinnGen Study; Steering Committee (University of Helsinki & Biobanks)

Supplemental Online Content: Nonauthor Collaborators
*First name, last name, and suffix (if applicable) are required and will appear in PubMed.

***First Name and Middle Initial(s)**	***Last Name**	***Suffix (eg, Jr, III)**	Academic Degrees	Institution	Location (city, state/province, country)	Role or Contribution, eg, chair, principal investigator	Group (if more than 1 Group listed in the byline) and/or Subgroup (eg, Steering Committee)
Jaakko	Kaprio			Institute for Molecular Medicine Finland (FIMM), HiLIFE, University of Helsinki	Helsinki, Finland		FinnGen Study; Steering Committee (University of Helsinki & Biobanks)
Minna	Ruddock			Arctic biobank / University of Oulu			FinnGen Study; Steering Committee (University of Helsinki & Biobanks)
Lila	Kallio			Auria Biobank / University of Turku / Wellbeing Services County of Southwest Finland	Turku, Finland		FinnGen Study; Steering Committee (University of Helsinki & Biobanks); Biobank directors
Antti	Hakanen			Auria Biobank / University of Turku / Wellbeing Services County of Southwest Finland	Turku, Finland		FinnGen Study; Steering Committee (University of Helsinki & Biobanks)
Terhi	Kilpi			THL Biobank / Finnish Institute for Health and Welfare (THL)	Helsinki, Finland		FinnGen Study; Steering Committee (University of Helsinki & Biobanks)
Markus	Perola			THL Biobank / Finnish Institute for Health and Welfare (THL)	Helsinki, Finland		FinnGen Study; Steering Committee (University of Helsinki & Biobanks); Scientific Committee (University of Helsinki & Biobanks)

*First name, last name, and suffix (if applicable) are required and will appear in PubMed.

***First Name and Middle Initial(s)**	***Last Name**	***Suffix (eg, Jr, III)**	Academic Degrees	Institution	Location (city, state/province, country)	Role or Contribution, eg, chair, principal investigator	Group (if more than 1 Group listed in the byline) and/or Subgroup (eg, Steering Committee)
Jukka	Partanen			Finnish Red Cross Blood Service / Finnish Hematology Registry and Clinical Biobank	Helsinki, Finland		FinnGen Study; Steering Committee (University of Helsinki & Biobanks); Biobank directors
Taneli	Raivio			Helsinki Biobank / Helsinki University and Hospital District of Helsinki and Uusimaa	Helsinki		FinnGen Study; Steering Committee (University of Helsinki & Biobanks)
Eero	Punkka			Helsinki Biobank / Helsinki University and Hospital District of Helsinki and Uusimaa	Helsinki		FinnGen Study; Steering Committee (University of Helsinki & Biobanks); Biobank directors
Teija	Kekonen			Northern Finland Biobank Borealis / University of Oulu / Wellbeing services county of North Ostrobothnia	Oulu, Finland		FinnGen Study; Steering Committee (University of Helsinki & Biobanks)
Raisa	Serpi			Northern Finland Biobank Borealis / University of Oulu / Wellbeing services county of North Ostrobothnia	Oulu, Finland		FinnGen Study; Steering Committee (University of Helsinki & Biobanks); Biobank directors
Kati	Kristiansson			Finnish Clinical Biobank Tampere / University of Tampere / Wellbeing Services County of Pirkanmaa	Tampere, Finland		FinnGen Study; Steering Committee (University of Helsinki & Biobanks)
Sanna	Siltanen			Finnish Clinical Biobank Tampere / University of Tampere / Wellbeing Services County of Pirkanmaa	Tampere, Finland		FinnGen Study; Steering Committee (University of Helsinki & Biobanks); Biobank directors

Supplemental Online Content: Nonauthor Collaborators
*First name, last name, and suffix (if applicable) are required and will appear in PubMed.

***First Name and Middle Initial(s)**	***Last Name**	***Suffix (eg, Jr, III)**	Academic Degrees	Institution	Location (city, state/province, country)	Role or Contribution, eg, chair, principal investigator	Group (if more than 1 Group listed in the byline) and/or Subgroup (eg, Steering Committee)
Veli-Matti	Kosma			Biobank of Eastern Finland			FinnGen Study; Steering Committee (University of Helsinki & Biobanks); Biobank directors
Arto	Mannermaa			Biobank of Eastern Finland			FinnGen Study; Steering Committee (University of Helsinki & Biobanks); Scientific Committee (University of Helsinki & Biobanks)
Jari	Laukkanen			Central Finland Biobank / University of Jyväskylä / Wellbeing Services County of Central Finland	Jyväskylä, Finland		FinnGen Study; Steering Committee (University of Helsinki & Biobanks); Scientific Committee (University of Helsinki & Biobanks)
Tiina	Jokela			Central Finland Biobank / University of Jyväskylä / Wellbeing Services County of Central Finland	Jyväskylä, Finland		FinnGen Study; Steering Committee (University of Helsinki & Biobanks); Biobank directors
Mervi	Ahlroth			Finnish Biobank Cooperative - FINBB			FinnGen Study; Steering Committee (University of Helsinki & Biobanks); FinnGen Teams (FINBB - Finnish biobank cooperative)

*First name, last name, and suffix (if applicable) are required and will appear in PubMed.

***First Name and Middle Initial(s)**	***Last Name**	***Suffix (eg, Jr, III)**	Academic Degrees	Institution	Location (city, state/province, country)	Role or Contribution, eg, chair, principal investigator	Group (if more than 1 Group listed in the byline) and/or Subgroup (eg, Steering Committee)
Johanna	Mäkelä			Finnish Biobank Cooperative - FINBB			FinnGen Study; Steering Committee (University of Helsinki & Biobanks); Scientific Committee (University of Helsinki & Biobanks); FinnGen Teams (FINBB - Finnish biobank cooperative)
Outi	Tuovila			Business Finland	Helsinki, Finland		FinnGen Study; Steering Committee (Other Experts/ Non-Voting Members)
Jeffrey	Waring			Abbvie	Chicago, IL, United States		FinnGen Study; Scientific Committee (Pharmaceutical companies)
Bridget	Riley-Gillis			Abbvie	Chicago, IL, United States		FinnGen Study; Scientific Committee (Pharmaceutical companies)
Fedik	Rahimov			Abbvie	Chicago, IL, United States		FinnGen Study; Scientific Committee (Pharmaceutical companies)
Ioanna	Tachmazidou			AstraZeneca	Cambridge, United Kingdom		FinnGen Study; Scientific Committee (Pharmaceutical companies)

*First name, last name, and suffix (if applicable) are required and will appear in PubMed.

***First Name and Middle Initial(s)**	***Last Name**	***Suffix (eg, Jr, III)**	Academic Degrees	Institution	Location (city, state/province, country)	Role or Contribution, eg, chair, principal investigator	Group (if more than 1 Group listed in the byline) and/or Subgroup (eg, Steering Committee)
Marc	Jung			Boehringer Ingelheim	Ingelheim am Rhein, Germany		FinnGen Study; Scientific Committee (Pharmaceutical companies)
Hanati	Tuoken			Boehringer Ingelheim	Ingelheim am Rhein, Germany		FinnGen Study; Scientific Committee (Pharmaceutical companies)
Shameek	Biswas			Bristol Myers Squibb	New York, NY, United States		FinnGen Study; Scientific Committee (Pharmaceutical companies)
Benjamin	Sun			Bristol Myers Squibb	New York, NY, United States		FinnGen Study; Scientific Committee (Pharmaceutical companies)
Neha	Raghavan			Merck	Kenilworth, NJ, United States		FinnGen Study; Scientific Committee (Pharmaceutical companies)
Jae-Hoon	Sul			Merck	Kenilworth, NJ, United States		FinnGen Study; Scientific Committee (Pharmaceutical companies)
Xinli	Hu			Pfizer	New York, NY, United States		FinnGen Study; Scientific Committee (Pharmaceutical companies)
Ma´en	Obeidat			Novartis Institutes for BioMedical Research	Cambridge, MA, United States		FinnGen Study; Scientific Committee (Pharmaceutical companies)

Supplemental Online Content: Nonauthor Collaborators
*First name, last name, and suffix (if applicable) are required and will appear in PubMed.

***First Name and Middle Initial(s)**	***Last Name**	***Suffix (eg, Jr, III)**	Academic Degrees	Institution	Location (city, state/province, country)	Role or Contribution, eg, chair, principal investigator	Group (if more than 1 Group listed in the byline) and/or Subgroup (eg, Steering Committee)
Jonathan	Chung			Novartis Institutes for BioMedical Research	Cambridge, MA, United States		FinnGen Study; Scientific Committee (Pharmaceutical companies)
Jonas	Zierer			Novartis Institutes for BioMedical Research	Cambridge, MA, United States		FinnGen Study; Scientific Committee (Pharmaceutical companies)
Mari	Niemi			Novartis Institutes for BioMedical Research	Cambridge, MA, United States		FinnGen Study; Scientific Committee (Pharmaceutical companies)
Samuli	Ripatti			Institute for Molecular Medicine Finland (FIMM), HiLIFE, University of Helsinki	Helsinki, Finland		FinnGen Study; Scientific Committee (University of Helsinki & Biobanks)
Johanna	Schleutker			Auria Biobank / University of Turku / Wellbeing Services County of Southwest Finland	Turku, Finland		FinnGen Study; Scientific Committee (University of Helsinki & Biobanks)
Tiina	Wahlfors			THL Biobank / Finnish Institute for Health and Welfare (THL)	Helsinki, Finland		FinnGen Study; Scientific Committee (University of Helsinki & Biobanks); Biobank directors
Mikko	Arvas			Finnish Red Cross Blood Service / Finnish Hematology Registry and Clinical Biobank	Helsinki, Finland		FinnGen Study; Scientific Committee (University of Helsinki & Biobanks)

Supplemental Online Content: Nonauthor Collaborators
*First name, last name, and suffix (if applicable) are required and will appear in PubMed.

***First Name and Middle Initial(s)**	***Last Name**	***Suffix (eg, Jr, III)**	Academic Degrees	Institution	Location (city, state/province, country)	Role or Contribution, eg, chair, principal investigator	Group (if more than 1 Group listed in the byline) and/or Subgroup (eg, Steering Committee)
Olli	Carpén			Helsinki Biobank / Helsinki University and Hospital District of Helsinki and Uusimaa	Helsinki		FinnGen Study; Scientific Committee (University of Helsinki & Biobanks)
Reetta	Hinttala			Northern Finland Biobank Borealis / University of Oulu / Wellbeing services county of North Ostrobothnia	Oulu, Finland		FinnGen Study; Scientific Committee (University of Helsinki & Biobanks)
Johannes	Kettunen			Northern Finland Biobank Borealis / University of Oulu / Wellbeing services county of North Ostrobothnia	Oulu, Finland		FinnGen Study; Scientific Committee (University of Helsinki & Biobanks)
Katriina	Aalto-Setälä			Faculty of Medicine and Health Technology, Tampere University	Tampere, Finland		FinnGen Study; Scientific Committee (University of Helsinki & Biobanks)
Mika	Kähönen			Finnish Clinical Biobank Tampere / University of Tampere / Wellbeing Services County of Pirkanmaa	Tampere, Finland		FinnGen Study; Scientific Committee (University of Helsinki & Biobanks); Clinical Group / Task Force
Hanna	Kujala			Biobank of Eastern Finland			FinnGen Study; Clinical Group / Task Force
Triin	Laisk			Estonian biobank	Tartu, Estonia		FinnGen Study; Clinical Group / Task Force
Natalia	Pujol			Estonian biobank	Tartu, Estonia		FinnGen Study; Clinical Group / Task Force

Supplemental Online Content: Nonauthor Collaborators
*First name, last name, and suffix (if applicable) are required and will appear in PubMed.

***First Name and Middle Initial(s)**	***Last Name**	***Suffix (eg, Jr, III)**	Academic Degrees	Institution	Location (city, state/province, country)	Role or Contribution, eg, chair, principal investigator	Group (if more than 1 Group listed in the byline) and/or Subgroup (eg, Steering Committee)
Veikko	Salomaa			Finnish Institute for Health and Welfare (THL)	Helsinki, Finland		FinnGen Study; Clinical Group / Task Force
Jaana	Suvisaari			Finnish Institute for Health and Welfare (THL)	Helsinki, Finland		FinnGen Study; Clinical Group / Task Force
Satu	Koskela			Finnish Red Cross Blood Service / Finnish Hematology Registry and Clinical Biobank	Helsinki, Finland		FinnGen Study; Clinical Group / Task Force
Jouni	Lauronen			Finnish Red Cross Blood Service / Finnish Hematology Registry and Clinical Biobank	Helsinki, Finland		FinnGen Study; Clinical Group / Task Force
Kristiina	Aittomäki			Helsinki University Central Hospital	Helsinki, Finland		FinnGen Study; Clinical Group / Task Force
Pirkko	Pussinen			Helsinki University Hospital and University of Helsinki	Helsinki, Finland		FinnGen Study; Clinical Group / Task Force
Tuomo	Meretoja			Helsinki University Hospital and University of Helsinki	Helsinki, Finland		FinnGen Study; Clinical Group / Task Force
Heikki	Joensuu			Helsinki University Hospital and University of Helsinki	Helsinki, Finland		FinnGen Study; Clinical Group / Task Force
Peeter	Karihtala			Helsinki University Hospital and University of Helsinki	Helsinki, Finland		FinnGen Study; Clinical Group / Task Force
Emma	Juuri			Helsinki University Hospital and University of Helsinki	Helsinki, Finland		FinnGen Study; Clinical Group / Task Force

Supplemental Online Content: Nonauthor Collaborators
*First name, last name, and suffix (if applicable) are required and will appear in PubMed.

***First Name and Middle Initial(s)**	***Last Name**	***Suffix (eg, Jr, III)**	Academic Degrees	Institution	Location (city, state/province, country)	Role or Contribution, eg, chair, principal investigator	Group (if more than 1 Group listed in the byline) and/or Subgroup (eg, Steering Committee)
Aino	Salminen			Helsinki University Hospital and University of Helsinki	Helsinki, Finland		FinnGen Study; Clinical Group / Task Force
Tuula	Salo			Helsinki University Hospital and University of Helsinki	Helsinki, Finland		FinnGen Study; Clinical Group / Task Force
David	Rice			Helsinki University Hospital and University of Helsinki	Helsinki, Finland		FinnGen Study; Clinical Group / Task Force
Pekka	Nieminen			Helsinki University Hospital and University of Helsinki	Helsinki, Finland		FinnGen Study; Clinical Group / Task Force
Ulla	Palotie			Helsinki University Hospital and University of Helsinki	Helsinki, Finland		FinnGen Study; Clinical Group / Task Force
Fredrik	Åberg			Helsinki University Hospital and University of Helsinki	Helsinki, Finland		FinnGen Study; Clinical Group / Task Force
Daniel	Gordin			Helsinki University Hospital and University of Helsinki	Helsinki, Finland		FinnGen Study; Clinical Group / Task Force
Patrik	Finne			Helsinki University Hospital and University of Helsinki	Helsinki, Finland		FinnGen Study; Clinical Group / Task Force
Minna	Raivio			Hospital District of Helsinki and Uusimaa	Helsinki, Finland		FinnGen Study; Clinical Group / Task Force
Pentti	Tienari			Hospital District of Helsinki and Uusimaa	Helsinki, Finland		FinnGen Study; Clinical Group / Task Force

Supplemental Online Content: Nonauthor Collaborators
*First name, last name, and suffix (if applicable) are required and will appear in PubMed.

***First Name and Middle Initial(s)**	***Last Name**	***Suffix (eg, Jr, III)**	Academic Degrees	Institution	Location (city, state/province, country)	Role or Contribution, eg, chair, principal investigator	Group (if more than 1 Group listed in the byline) and/or Subgroup (eg, Steering Committee)
Martti	Färkkilä			Hospital District of Helsinki and Uusimaa	Helsinki, Finland		FinnGen Study; Clinical Group / Task Force
Jukka	Koskela			Hospital District of Helsinki and Uusimaa	Helsinki, Finland		FinnGen Study; Clinical Group / Task Force
Sampsa	Pikkarainen			Hospital District of Helsinki and Uusimaa	Helsinki, Finland		FinnGen Study; Clinical Group / Task Force
Kari	Eklund			Hospital District of Helsinki and Uusimaa	Helsinki, Finland		FinnGen Study; Clinical Group / Task Force
Paula	Kauppi			Hospital District of Helsinki and Uusimaa	Helsinki, Finland		FinnGen Study; Clinical Group / Task Force
Juha	Sinisalo			Hospital District of Helsinki and Uusimaa	Helsinki, Finland		FinnGen Study; Clinical Group / Task Force
Marja-Riitta	Taskinen			Hospital District of Helsinki and Uusimaa	Helsinki, Finland		FinnGen Study; Clinical Group / Task Force
Tiinamaija	Tuomi			Hospital District of Helsinki and Uusimaa	Helsinki, Finland		FinnGen Study; Clinical Group / Task Force
Timo	Hiltunen			Hospital District of Helsinki and Uusimaa	Helsinki, Finland		FinnGen Study; Clinical Group / Task Force
Johanna	Mattson			Hospital District of Helsinki and Uusimaa	Helsinki, Finland		FinnGen Study; Clinical Group / Task Force

Supplemental Online Content: Nonauthor Collaborators
*First name, last name, and suffix (if applicable) are required and will appear in PubMed.

***First Name and Middle Initial(s)**	***Last Name**	***Suffix (eg, Jr, III)**	Academic Degrees	Institution	Location (city, state/province, country)	Role or Contribution, eg, chair, principal investigator	Group (if more than 1 Group listed in the byline) and/or Subgroup (eg, Steering Committee)
Eveliina	Salminen			Hospital District of Helsinki and Uusimaa	Helsinki, Finland		FinnGen Study; Clinical Group / Task Force
Terhi	Ollila			Hospital District of Helsinki and Uusimaa	Helsinki, Finland		FinnGen Study; Clinical Group / Task Force
Katariina	Hannula-Jouppi			Hospital District of Helsinki and Uusimaa	Helsinki, Finland		FinnGen Study; Clinical Group / Task Force
Oskari	Heikinheimo			Hospital District of Helsinki and Uusimaa	Helsinki, Finland		FinnGen Study; Clinical Group / Task Force
Ilkka	Kalliala			Hospital District of Helsinki and Uusimaa	Helsinki, Finland		FinnGen Study; Clinical Group / Task Force
Lauri	Aaltonen			Hospital District of Helsinki and Uusimaa	Helsinki, Finland		FinnGen Study; Clinical Group / Task Force
Erkki	Isometsä			Hospital District of Helsinki and Uusimaa	Helsinki, Finland		FinnGen Study; Clinical Group / Task Force
Antti	Aarnisalo			Hospital District of Helsinki and Uusimaa	Helsinki, Finland		FinnGen Study; Clinical Group / Task Force
Ilkka	Immonen			Hospital District of Helsinki and Uusimaa	Helsinki, Finland		FinnGen Study; Clinical Group / Task Force
Salla	Ranta			Hospital District of Helsinki and Uusimaa	Helsinki, Finland		FinnGen Study; Clinical Group / Task Force

Supplemental Online Content: Nonauthor Collaborators
*First name, last name, and suffix (if applicable) are required and will appear in PubMed.

***First Name and Middle Initial(s)**	***Last Name**	***Suffix (eg, Jr, III)**	Academic Degrees	Institution	Location (city, state/province, country)	Role or Contribution, eg, chair, principal investigator	Group (if more than 1 Group listed in the byline) and/or Subgroup (eg, Steering Committee)
Filip	Scheperjans			Hospital District of Helsinki and Uusimaa	Helsinki, Finland		FinnGen Study; Clinical Group / Task Force
Felix	Vaura			Institute for Molecular Medicine Finland (FIMM), HiLIFE, University of Helsinki	Helsinki, Finland		FinnGen Study; Clinical Group / Task Force
Nina	Mars			Institute for Molecular Medicine Finland (FIMM), HiLIFE, University of Helsinki	Helsinki, Finland		FinnGen Study; Clinical Group / Task Force
Esa	Pitkänen			Institute for Molecular Medicine Finland (FIMM), HiLIFE, University of Helsinki	Helsinki, Finland		FinnGen Study; Clinical Group / Task Force
Hannele	Laivuori			Institute for Molecular Medicine Finland (FIMM), HiLIFE, University of Helsinki	Helsinki, Finland		FinnGen Study; Clinical Group / Task Force
Tuomo	Kiiskinen			Institute for Molecular Medicine Finland (FIMM), HiLIFE, University of Helsinki	Helsinki, Finland		FinnGen Study; Clinical Group / Task Force
Katja	Kivinen			Institute for Molecular Medicine Finland (FIMM), HiLIFE, University of Helsinki	Helsinki, Finland		FinnGen Study; Clinical Group / Task Force
Elisabeth	Widen			Institute for Molecular Medicine Finland (FIMM), HiLIFE, University of Helsinki	Helsinki, Finland		FinnGen Study; Clinical Group / Task Force
Taru	Tukiainen			Institute for Molecular Medicine Finland (FIMM), HiLIFE, University of Helsinki	Helsinki, Finland		FinnGen Study; Clinical Group / Task Force
Hanna	Ollila			Institute for Molecular Medicine Finland (FIMM), HiLIFE, University of Helsinki	Helsinki, Finland		FinnGen Study; Clinical Group / Task Force

Supplemental Online Content: Nonauthor Collaborators
*First name, last name, and suffix (if applicable) are required and will appear in PubMed.

***First Name and Middle Initial(s)**	***Last Name**	***Suffix (eg, Jr, III)**	Academic Degrees	Institution	Location (city, state/province, country)	Role or Contribution, eg, chair, principal investigator	Group (if more than 1 Group listed in the byline) and/or Subgroup (eg, Steering Committee)
Elmo	Saarentaus			Institute for Molecular Medicine Finland (FIMM), HiLIFE, University of Helsinki	Helsinki, Finland		FinnGen Study; Clinical Group / Task Force
Anne	Kerola			Institute for Molecular Medicine Finland (FIMM), HiLIFE, University of Helsinki	Helsinki, Finland		FinnGen Study; Clinical Group / Task Force
Eero	Vuoksimaa			Institute for Molecular Medicine Finland (FIMM), HiLIFE, University of Helsinki	Helsinki, Finland		FinnGen Study; Clinical Group / Task Force
Joni	Lindbohm			Institute for Molecular Medicine Finland (FIMM), HiLIFE, University of Helsinki	Helsinki, Finland		FinnGen Study; Clinical Group / Task Force
Zhiyu	Yang			Institute for Molecular Medicine Finland (FIMM), HiLIFE, University of Helsinki	Helsinki, Finland		FinnGen Study; Clinical Group / Task Force
Matthew	Sampson			Institute for Molecular Medicine Finland (FIMM), HiLIFE, University of Helsinki	Helsinki, Finland		FinnGen Study; Clinical Group / Task Force
Michelle	McNulty			Institute for Molecular Medicine Finland (FIMM), HiLIFE, University of Helsinki	Helsinki, Finland		FinnGen Study; Clinical Group / Task Force
Aoxing	Liu			Institute for Molecular Medicine Finland (FIMM), HiLIFE, University of Helsinki	Helsinki, Finland		FinnGen Study; Clinical Group / Task Force
Austin	Argentieri			Institute for Molecular Medicine Finland (FIMM), HiLIFE, University of Helsinki	Helsinki, Finland		FinnGen Study; Clinical Group / Task Force
Amanda	Elliott			Institute for Molecular Medicine Finland (FIMM), HiLIFE, University of Helsinki	Helsinki, Finland		FinnGen Study; Clinical Group / Task Force

Supplemental Online Content: Nonauthor Collaborators
*First name, last name, and suffix (if applicable) are required and will appear in PubMed.

***First Name and Middle Initial(s)**	***Last Name**	***Suffix (eg, Jr, III)**	Academic Degrees	Institution	Location (city, state/province, country)	Role or Contribution, eg, chair, principal investigator	Group (if more than 1 Group listed in the byline) and/or Subgroup (eg, Steering Committee)
Elisa	Rahikkala			Northern Ostrobothnia Hospital District	Oulu, Finland		FinnGen Study; Clinical Group / Task Force
Kirsi	Sipilä			Oulu University Hospital and University of Oulu	Oulu, Finland		FinnGen Study; Clinical Group / Task Force
Valtteri	Julkunen			University of Eastern Finland and Kuopio University Hospital	Kuopio, Finland		FinnGen Study; Clinical Group / Task Force
Ville	Leinonen			University of Eastern Finland and Kuopio University Hospital	Kuopio, Finland		FinnGen Study; Clinical Group / Task Force
Sanna	Toppila-Salmi			University of Eastern Finland and Kuopio University Hospital	Kuopio, Finland		FinnGen Study; Clinical Group / Task Force
Mikko	Hiltunen			University of Eastern Finland	Kuopio, Finland		FinnGen Study; Clinical Group / Task Force
Eino	Solje			University of Eastern Finland	Kuopio, Finland		FinnGen Study; Clinical Group / Task Force
Hannu	Kankaanranta			University of Gothenburg	Gothenburg, Sweden		FinnGen Study; Clinical Group / Task Force
Antti	Mäkitie			University of Helsinki and Helsinki University Hospital	Helsinki, Finland		FinnGen Study; Clinical Group / Task Force
Iiris	Hovatta			University of Helsinki	Helsinki, Finland		FinnGen Study; Clinical Group / Task Force

Supplemental Online Content: Nonauthor Collaborators
*First name, last name, and suffix (if applicable) are required and will appear in PubMed.

***First Name and Middle Initial(s)**	***Last Name**	***Suffix (eg, Jr, III)**	Academic Degrees	Institution	Location (city, state/province, country)	Role or Contribution, eg, chair, principal investigator	Group (if more than 1 Group listed in the byline) and/or Subgroup (eg, Steering Committee)
Niko	Välimäki			University of Helsinki	Helsinki, Finland		FinnGen Study; Clinical Group / Task Force
Minttu	Marttila			University of Helsinki	Helsinki, Finland		FinnGen Study; Clinical Group / Task Force
Anne	Portaankorva			University of Helsinki	Helsinki, Finland		FinnGen Study; Clinical Group / Task Force
Eija	Laakkonen			University of Jyväskylä	Jyväskylä, Finland		FinnGen Study; Clinical Group / Task Force
Heidi	Silven			University of Oulu	Oulu, Finland		FinnGen Study; Clinical Group / Task Force
Eeva	Sliz			University of Oulu	Oulu, Finland		FinnGen Study; Clinical Group / Task Force
Riikka	Arffman			University of Oulu	Oulu, Finland		FinnGen Study; Clinical Group / Task Force
Susanna	Savukoski			University of Oulu	Oulu, Finland		FinnGen Study; Clinical Group / Task Force
Riitta	Kaarteenaho			University of Oulu	Oulu, Finland		FinnGen Study; Clinical Group / Task Force
Minna	Karjalainen			University of Oulu	Oulu, Finland		FinnGen Study; Clinical Group / Task Force

*First name, last name, and suffix (if applicable) are required and will appear in PubMed.

***First Name and Middle Initial(s)**	***Last Name**	***Suffix (eg, Jr, III)**	Academic Degrees	Institution	Location (city, state/province, country)	Role or Contribution, eg, chair, principal investigator	Group (if more than 1 Group listed in the byline) and/or Subgroup (eg, Steering Committee)
Jaakko	Tyrmi			University of Oulu	Oulu, Finland		FinnGen Study; Clinical Group / Task Force
Laura	Kuusalo			University of Turku	Turku, Finland		FinnGen Study; Clinical Group / Task Force
Laura	Pirilä			University of Turku	Turku, Finland		FinnGen Study; Clinical Group / Task Force
Tapio	Hellman			University of Turku	Turku, Finland		FinnGen Study; Clinical Group / Task Force
Matti	Vuori			University of Turku	Turku, Finland		FinnGen Study; Clinical Group / Task Force
Teemu	Niiranen			University of Turku	Turku, Finland		FinnGen Study; Clinical Group / Task Force
Timo	Blomster			Wellbeing services county of North Ostrobothnia	Oulu, Finland		FinnGen Study; Clinical Group / Task Force
Johanna	Huhtakangas			Wellbeing services county of North Ostrobothnia	Oulu, Finland		FinnGen Study; Clinical Group / Task Force
Terttu	Harju			Wellbeing services county of North Ostrobothnia	Oulu, Finland		FinnGen Study; Clinical Group / Task Force
Kaisa	Tasanen			Wellbeing services county of North Ostrobothnia	Oulu, Finland		FinnGen Study; Clinical Group / Task Force

Supplemental Online Content: Nonauthor Collaborators
*First name, last name, and suffix (if applicable) are required and will appear in PubMed.

***First Name and Middle Initial(s)**	***Last Name**	***Suffix (eg, Jr, III)**	Academic Degrees	Institution	Location (city, state/province, country)	Role or Contribution, eg, chair, principal investigator	Group (if more than 1 Group listed in the byline) and/or Subgroup (eg, Steering Committee)
Laura	Huilaja			Wellbeing services county of North Ostrobothnia	Oulu, Finland		FinnGen Study; Clinical Group / Task Force
Vuokko	Anttonen			Wellbeing services county of North Ostrobothnia	Oulu, Finland		FinnGen Study; Clinical Group / Task Force
Marja	Vääräsmäki			Wellbeing services county of North Ostrobothnia	Oulu, Finland		FinnGen Study; Clinical Group / Task Force
Outi	Uimari			Wellbeing services county of North Ostrobothnia	Oulu, Finland		FinnGen Study; Clinical Group / Task Force
Laure	Morin-Papunen			Wellbeing services county of North Ostrobothnia	Oulu, Finland		FinnGen Study; Clinical Group / Task Force
Maarit	Niinimäki			Wellbeing services county of North Ostrobothnia	Oulu, Finland		FinnGen Study; Clinical Group / Task Force
Terhi	Piltonen			Wellbeing services county of North Ostrobothnia	Oulu, Finland		FinnGen Study; Clinical Group / Task Force
Reetta	Kälviäinen			Wellbeing services county of North Savo	Kuopio, Finland		FinnGen Study; Clinical Group / Task Force
Hilkka	Soininen			Wellbeing services county of North Savo	Kuopio, Finland		FinnGen Study; Clinical Group / Task Force
Mikko	Kiviniemi			Wellbeing services county of North Savo	Kuopio, Finland		FinnGen Study; Clinical Group / Task Force

Supplemental Online Content: Nonauthor Collaborators
*First name, last name, and suffix (if applicable) are required and will appear in PubMed.

***First Name and Middle Initial(s)**	***Last Name**	***Suffix (eg, Jr, III)**	Academic Degrees	Institution	Location (city, state/province, country)	Role or Contribution, eg, chair, principal investigator	Group (if more than 1 Group listed in the byline) and/or Subgroup (eg, Steering Committee)
Oili	Kaipiainen-Seppänen			Wellbeing services county of North Savo	Kuopio, Finland		FinnGen Study; Clinical Group / Task Force
Margit	Pelkonen			Wellbeing services county of North Savo	Kuopio, Finland		FinnGen Study; Clinical Group / Task Force
Päivi	Auvinen			Wellbeing services county of North Savo	Kuopio, Finland		FinnGen Study; Clinical Group / Task Force
Maria	Siponen			Wellbeing services county of North Savo	Kuopio, Finland		FinnGen Study; Clinical Group / Task Force
Liisa	Suominen			Wellbeing services county of North Savo	Kuopio, Finland		FinnGen Study; Clinical Group / Task Force
Päivi	Mäntylä			Wellbeing services county of North Savo	Kuopio, Finland		FinnGen Study; Clinical Group / Task Force
Jukka	Peltola			Wellbeing Services County of Pirkanmaa	Tampere, Finland		FinnGen Study; Clinical Group / Task Force
Airi	Jussila			Wellbeing Services County of Pirkanmaa	Tampere, Finland		FinnGen Study; Clinical Group / Task Force
Katri	Kaukinen			Wellbeing Services County of Pirkanmaa	Tampere, Finland		FinnGen Study; Clinical Group / Task Force
Pia	Isomäki			Wellbeing Services County of Pirkanmaa	Tampere, Finland		FinnGen Study; Clinical Group / Task Force

*First name, last name, and suffix (if applicable) are required and will appear in PubMed.

***First Name and Middle Initial(s)**	***Last Name**	***Suffix (eg, Jr, III)**	Academic Degrees	Institution	Location (city, state/province, country)	Role or Contribution, eg, chair, principal investigator	Group (if more than 1 Group listed in the byline) and/or Subgroup (eg, Steering Committee)
Jussi	Hernesniemi			Wellbeing Services County of Pirkanmaa	Tampere, Finland		FinnGen Study; Clinical Group / Task Force
Annika	Auranen			Wellbeing Services County of Pirkanmaa	Tampere, Finland		FinnGen Study; Clinical Group / Task Force
Hannu	Uusitalo			Wellbeing Services County of Pirkanmaa	Tampere, Finland		FinnGen Study; Clinical Group / Task Force
Teea	Salmi			Wellbeing Services County of Pirkanmaa	Tampere, Finland		FinnGen Study; Clinical Group / Task Force
Venla	Kurra			Wellbeing Services County of Pirkanmaa	Tampere, Finland		FinnGen Study; Clinical Group / Task Force
Laura	Kotaniemi-Talonen			Wellbeing Services County of Pirkanmaa	Tampere, Finland		FinnGen Study; Clinical Group / Task Force
Argyro	Bizaki-Vallaskangas			Wellbeing Services County of Pirkanmaa	Tampere, Finland		FinnGen Study; Clinical Group / Task Force
Juha	Rinne			Wellbeing Services County of Southwest Finland	Turku, Finland		FinnGen Study; Clinical Group / Task Force
Roosa	Kallionpää			Wellbeing Services County of Southwest Finland	Turku, Finland		FinnGen Study; Clinical Group / Task Force
Markku	Voutilainen			Wellbeing Services County of Southwest Finland	Turku, Finland		FinnGen Study; Clinical Group / Task Force

Supplemental Online Content: Nonauthor Collaborators
*First name, last name, and suffix (if applicable) are required and will appear in PubMed.

***First Name and Middle Initial(s)**	***Last Name**	***Suffix (eg, Jr, III)**	Academic Degrees	Institution	Location (city, state/province, country)	Role or Contribution, eg, chair, principal investigator	Group (if more than 1 Group listed in the byline) and/or Subgroup (eg, Steering Committee)
Antti	Palomäki			Wellbeing Services County of Southwest Finland	Turku, Finland		FinnGen Study; Clinical Group / Task Force
Riitta	Lahesmaa			Wellbeing Services County of Southwest Finland	Turku, Finland		FinnGen Study; Clinical Group / Task Force
Kaj	Metsärinne			Wellbeing Services County of Southwest Finland	Turku, Finland		FinnGen Study; Clinical Group / Task Force
Jenni	Aittokallio			Wellbeing Services County of Southwest Finland	Turku, Finland		FinnGen Study; Clinical Group / Task Force
Klaus	Elenius			Wellbeing Services County of Southwest Finland	Turku, Finland		FinnGen Study; Clinical Group / Task Force
Sirkku	Peltonen			Wellbeing Services County of Southwest Finland	Turku, Finland		FinnGen Study; Clinical Group / Task Force
Leena	Koulu			Wellbeing Services County of Southwest Finland	Turku, Finland		FinnGen Study; Clinical Group / Task Force
Ulvi	Gursoy			Wellbeing Services County of Southwest Finland	Turku, Finland		FinnGen Study; Clinical Group / Task Force
Varpu	Jokimaa			Wellbeing Services County of Southwest Finland	Turku, Finland		FinnGen Study; Clinical Group / Task Force
Tytti	Willberg			Wellbeing Services County of Southwest Finland	Turku, Finland		FinnGen Study; Clinical Group / Task Force

*First name, last name, and suffix (if applicable) are required and will appear in PubMed.

***First Name and Middle Initial(s)**	***Last Name**	***Suffix (eg, Jr, III)**	Academic Degrees	Institution	Location (city, state/province, country)	Role or Contribution, eg, chair, principal investigator	Group (if more than 1 Group listed in the byline) and/or Subgroup (eg, Steering Committee)
Adam	Ziemann			Abbvie	Chicago, IL, United States		FinnGen Study; Clinical Group / Task Force
Nizar	Smaoui			Abbvie	Chicago, IL, United States		FinnGen Study; Clinical Group / Task Force
Anne	Lehtonen			Abbvie	Chicago, IL, United States		FinnGen Study; Clinical Group / Task Force
Apinya	Lertratanakul			Abbvie	Chicago, IL, United States		FinnGen Study; Clinical Group / Task Force
Relja	Popovic			Abbvie	Chicago, IL, United States		FinnGen Study; Clinical Group / Task Force
Mengzhen	Liu			Abbvie	Chicago, IL, United States		FinnGen Study; Clinical Group / Task Force
Anneke	Den Hollander			AbbVie	Chicago, IL, United States		FinnGen Study; Clinical Group / Task Force
Jan	Freudenberg			AbbVie	Chicago, IL, United States		FinnGen Study; Clinical Group / Task Force
Britney	Milkovich			AbbVie	Chicago, IL, United States		FinnGen Study; Clinical Group / Task Force
Andrew	Blumenfeld			AbbVie	Chicago, IL, United States		FinnGen Study; Clinical Group / Task Force

Supplemental Online Content: Nonauthor Collaborators
*First name, last name, and suffix (if applicable) are required and will appear in PubMed.

***First Name and Middle Initial(s)**	***Last Name**	***Suffix (eg, Jr, III)**	Academic Degrees	Institution	Location (city, state/province, country)	Role or Contribution, eg, chair, principal investigator	Group (if more than 1 Group listed in the byline) and/or Subgroup (eg, Steering Committee)
Tushar	Kumar			AbbVie	Chicago, IL, United States		FinnGen Study; Clinical Group / Task Force
Dirk	Paul			AstraZeneca	Cambridge, United Kingdom		FinnGen Study; Clinical Group / Task Force
Bram	Prins			AstraZeneca	Cambridge, United Kingdom		FinnGen Study; Clinical Group / Task Force
Eleanor	Wheeler			AstraZeneca	Cambridge, United Kingdom		FinnGen Study; Clinical Group / Task Force
Kousik	Kundu			AstraZeneca	Cambridge, United Kingdom		FinnGen Study; Clinical Group / Task Force
Santosh	Atanur			AstraZeneca	Cambridge, United Kingdom		FinnGen Study; Clinical Group / Task Force
Andrew	Lowe			AstraZeneca	Cambridge, United Kingdom		FinnGen Study; Clinical Group / Task Force
Thomas	Spargo			AstraZeneca	Cambridge, United Kingdom		FinnGen Study; Clinical Group / Task Force
Oliver	Burren			AstraZeneca	Cambridge, United Kingdom		FinnGen Study; Clinical Group / Task Force
Margarete	Fabre			AstraZeneca	Cambridge, United Kingdom		FinnGen Study; Clinical Group / Task Force

Supplemental Online Content: Nonauthor Collaborators
*First name, last name, and suffix (if applicable) are required and will appear in PubMed.

***First Name and Middle Initial(s)**	***Last Name**	***Suffix (eg, Jr, III)**	Academic Degrees	Institution	Location (city, state/province, country)	Role or Contribution, eg, chair, principal investigator	Group (if more than 1 Group listed in the byline) and/or Subgroup (eg, Steering Committee)
Fabio	Baschiera			Bayer AG	Leverkusen, Germany		FinnGen Study; Clinical Group / Task Force
Hans	van Leeuwen			Bayer AG	Leverkusen, Germany		FinnGen Study; Clinical Group / Task Force
Himanshu	Manchanda			Bayer AG	Leverkusen, Germany		FinnGen Study; Clinical Group / Task Force
Karl	Heilbron			Bayer AG	Leverkusen, Germany		FinnGen Study; Clinical Group / Task Force
Martin	Rao			Bayer AG	Leverkusen, Germany		FinnGen Study; Clinical Group / Task Force
Nicole	Schmidt			Bayer AG	Leverkusen, Germany		FinnGen Study; Clinical Group / Task Force
Samu	Kurki			Bayer AG	Leverkusen, Germany		FinnGen Study; Clinical Group / Task Force
Johanna	Mielke			Bayer AG	Leverkusen, Germany		FinnGen Study; Clinical Group / Task Force
Juho	Immonen			Bayer AG	Leverkusen, Germany		FinnGen Study; Clinical Group / Task Force
Thomas	Battram			Bayer AG	Leverkusen, Germany		FinnGen Study; Clinical Group / Task Force

Supplemental Online Content: Nonauthor Collaborators
*First name, last name, and suffix (if applicable) are required and will appear in PubMed.

***First Name and Middle Initial(s)**	***Last Name**	***Suffix (eg, Jr, III)**	Academic Degrees	Institution	Location (city, state/province, country)	Role or Contribution, eg, chair, principal investigator	Group (if more than 1 Group listed in the byline) and/or Subgroup (eg, Steering Committee)
Tobias	Hogrebe			Bayer AG	Leverkusen, Germany		FinnGen Study; Clinical Group / Task Force
Susan	Eaton			Biogen	Cambridge, MA, United States		FinnGen Study; Clinical Group / Task Force
Ketian	Yu			Biogen	Cambridge, MA, United States		FinnGen Study; Clinical Group / Task Force
Stephanie	Loomis			Biogen	Cambridge, MA, United States		FinnGen Study; Clinical Group / Task Force
Coro	Paisan-Ruiz			Biogen	Cambridge, MA, United States		FinnGen Study; Clinical Group / Task Force
Elke	Markert			Boehringer Ingelheim	Ingelheim am Rhein, Germany		FinnGen Study; Clinical Group / Task Force
Frank	Li			Boehringer Ingelheim	Ingelheim am Rhein, Germany		FinnGen Study; Clinical Group / Task Force
Yao	Hu			Boehringer Ingelheim	Ingelheim am Rhein, Germany		FinnGen Study; Clinical Group / Task Force
Christoph	Ogris			Boehringer Ingelheim	Ingelheim am Rhein, Germany		FinnGen Study; Clinical Group / Task Force
Eric	Simon			Boehringer Ingelheim	Ingelheim am Rhein, Germany		FinnGen Study; Clinical Group / Task Force

Supplemental Online Content: Nonauthor Collaborators
*First name, last name, and suffix (if applicable) are required and will appear in PubMed.

***First Name and Middle Initial(s)**	***Last Name**	***Suffix (eg, Jr, III)**	Academic Degrees	Institution	Location (city, state/province, country)	Role or Contribution, eg, chair, principal investigator	Group (if more than 1 Group listed in the byline) and/or Subgroup (eg, Steering Committee)
Julio Cesar	Bolivar Lopez			Boehringer Ingelheim	Ingelheim am Rhein, Germany		FinnGen Study; Clinical Group / Task Force
Monika	Frysz			Boehringer Ingelheim	Ingelheim am Rhein, Germany		FinnGen Study; Clinical Group / Task Force
Marla	Hochfeld			Bristol Myers Squibb	New York, NY, United States		FinnGen Study; Clinical Group / Task Force
Cara	Carty			Bristol Myers Squibb	New York, NY, United States		FinnGen Study; Clinical Group / Task Force
Michael	Turchin			Bristol Myers Squibb	New York, NY, United States		FinnGen Study; Clinical Group / Task Force
Neelakshi	Jog			Bristol Myers Squibb	New York, NY, United States		FinnGen Study; Clinical Group / Task Force
Corneliu	Bodea			Bristol Myers Squibb	New York, NY, United States		FinnGen Study; Clinical Group / Task Force
Janie	Shelton			Bristol Myers Squibb	New York, NY, United States		FinnGen Study; Clinical Group / Task Force
Chen	Li			Bristol Myers Squibb	New York, NY, United States		FinnGen Study; Clinical Group / Task Force
Kritika	Singh			Bristol Myers Squibb	New York, NY, United States		FinnGen Study; Clinical Group / Task Force

Supplemental Online Content: Nonauthor Collaborators
*First name, last name, and suffix (if applicable) are required and will appear in PubMed.

***First Name and Middle Initial(s)**	***Last Name**	***Suffix (eg, Jr, III)**	Academic Degrees	Institution	Location (city, state/province, country)	Role or Contribution, eg, chair, principal investigator	Group (if more than 1 Group listed in the byline) and/or Subgroup (eg, Steering Committee)
Peng	Jiang			Bristol Myers Squibb	New York, NY, United States		FinnGen Study; Clinical Group / Task Force
Elena	Sanchez			Bristol Myers Squibb	New York, NY, United States		FinnGen Study; Clinical Group / Task Force
Lilith	Moss			Bristol Myers Squibb	New York, NY, United States		FinnGen Study; Clinical Group / Task Force
Zijie	Zhao			Bristol Myers Squibb	New York, NY, United States		FinnGen Study; Clinical Group / Task Force
Anna	Podgornaia			Bristol Myers Squibb	New York, NY, United States		FinnGen Study; Clinical Group / Task Force
Natalie	Bowers			Genentech	San Francisco, CA, United States		FinnGen Study; Clinical Group / Task Force
Edmond	Teng			Genentech	San Francisco, CA, United States		FinnGen Study; Clinical Group / Task Force
Tim	Lu			Genentech	San Francisco, CA, United States		FinnGen Study; Clinical Group / Task Force
Hubert	Chen			Genentech	San Francisco, CA, United States		FinnGen Study; Clinical Group / Task Force
Jennifer	Schutzman			Genentech	San Francisco, CA, United States		FinnGen Study; Clinical Group / Task Force

Supplemental Online Content: Nonauthor Collaborators
*First name, last name, and suffix (if applicable) are required and will appear in PubMed.

***First Name and Middle Initial(s)**	***Last Name**	***Suffix (eg, Jr, III)**	Academic Degrees	Institution	Location (city, state/province, country)	Role or Contribution, eg, chair, principal investigator	Group (if more than 1 Group listed in the byline) and/or Subgroup (eg, Steering Committee)
Erich	Strauss			Genentech	San Francisco, CA, United States		FinnGen Study; Clinical Group / Task Force
Hao	Chen			Genentech	San Francisco, CA, United States		FinnGen Study; Clinical Group / Task Force
David	Choy			Genentech	San Francisco, CA, United States		FinnGen Study; Clinical Group / Task Force
Brian	Yaspan			Genentech	San Francisco, CA, United States		FinnGen Study; Clinical Group / Task Force
Cameron	Adams			Genentech	San Francisco, CA, United States		FinnGen Study; Clinical Group / Task Force
Michael	Rothenberg			Genentech	San Francisco, CA, United States		FinnGen Study; Clinical Group / Task Force
Sergio	Dellepiane			Genentech	San Francisco, CA, United States		FinnGen Study; Clinical Group / Task Force
Anubha	Mahajan			Genentech	San Francisco, CA, United States		FinnGen Study; Clinical Group / Task Force
Michael	Holmes			Genentech	San Francisco, CA, United States		FinnGen Study; Clinical Group / Task Force
Diana	Chang			Genentech	San Francisco, CA, United States		FinnGen Study; Clinical Group / Task Force

Supplemental Online Content: Nonauthor Collaborators
*First name, last name, and suffix (if applicable) are required and will appear in PubMed.

***First Name and Middle Initial(s)**	***Last Name**	***Suffix (eg, Jr, III)**	Academic Degrees	Institution	Location (city, state/province, country)	Role or Contribution, eg, chair, principal investigator	Group (if more than 1 Group listed in the byline) and/or Subgroup (eg, Steering Committee)
Tushar	Bhangale			Genentech	San Francisco, CA, United States		FinnGen Study; Clinical Group / Task Force
Fanli	Xu			GlaxoSmithKline	Brentford, United Kingdom		FinnGen Study; Clinical Group / Task Force
Laura	Addis			GlaxoSmithKline	Brentford, United Kingdom		FinnGen Study; Clinical Group / Task Force
John	Eicher			GlaxoSmithKline	Brentford, United Kingdom		FinnGen Study; Clinical Group / Task Force
Linda	McCarthy			GlaxoSmithKline	Brentford, United Kingdom		FinnGen Study; Clinical Group / Task Force
Jorge	Esparza Gordillo			GlaxoSmithKline	Brentford, United Kingdom		FinnGen Study; Clinical Group / Task Force
Joanna	Betts			GlaxoSmithKline	Brentford, United Kingdom		FinnGen Study; Clinical Group / Task Force
Rajashree	Mishra			GlaxoSmithKline	Brentford, United Kingdom		FinnGen Study; Clinical Group / Task Force
Audrey	Chu			GlaxoSmithKline	Brentford, United Kingdom		FinnGen Study; Clinical Group / Task Force
Diptee	Kulkarni			GlaxoSmithKline	Brentford, United Kingdom		FinnGen Study; Clinical Group / Task Force

Supplemental Online Content: Nonauthor Collaborators
*First name, last name, and suffix (if applicable) are required and will appear in PubMed.

***First Name and Middle Initial(s)**	***Last Name**	***Suffix (eg, Jr, III)**	Academic Degrees	Institution	Location (city, state/province, country)	Role or Contribution, eg, chair, principal investigator	Group (if more than 1 Group listed in the byline) and/or Subgroup (eg, Steering Committee)
Janet	Kumar			GlaxoSmithKline	Collegeville, PA, United States		FinnGen Study; Clinical Group / Task Force
Charli	Harlow			GlaxoSmithKline	Collegeville, PA, United States		FinnGen Study; Clinical Group / Task Force
Lea	Sarow-Blat			GlaxoSmithKline	Collegeville, PA, United States		FinnGen Study; Clinical Group / Task Force
Diana L.	Cousminer			GlaxoSmithKline	Collegeville, PA, United States		FinnGen Study; Clinical Group / Task Force
Jagtar	Nijjar			GlaxoSmithKline	Collegeville, PA, United States		FinnGen Study; Clinical Group / Task Force
Jessica	Chao			GlaxoSmithKline	Collegeville, PA, United States		FinnGen Study; Clinical Group / Task Force
Michal	Magid			GlaxoSmithKline	Collegeville, PA, United States		FinnGen Study; Clinical Group / Task Force
Shashank	Jariwala			GlaxoSmithKline	Collegeville, PA, United States		FinnGen Study; Clinical Group / Task Force
Chris	Floyd			GlaxoSmithKline	Collegeville, PA, United States		FinnGen Study; Clinical Group / Task Force
Dan	Swerdlow			GlaxoSmithKline	Collegeville, PA, United States		FinnGen Study; Clinical Group / Task Force

Supplemental Online Content: Nonauthor Collaborators
*First name, last name, and suffix (if applicable) are required and will appear in PubMed.

***First Name and Middle Initial(s)**	***Last Name**	***Suffix (eg, Jr, III)**	Academic Degrees	Institution	Location (city, state/province, country)	Role or Contribution, eg, chair, principal investigator	Group (if more than 1 Group listed in the byline) and/or Subgroup (eg, Steering Committee)
Erding	Hu			GlaxoSmithKline	Collegeville, PA, United States		FinnGen Study; Clinical Group / Task Force
Prerak	Desai			GlaxoSmithKline	Collegeville, PA, United States		FinnGen Study; Clinical Group / Task Force
Stephen	Haddad			GlaxoSmithKline	Collegeville, PA, United States		FinnGen Study; Clinical Group / Task Force
Damien	Croteau-Chonka			GlaxoSmithKline	Collegeville, PA, United States		FinnGen Study; Clinical Group / Task Force
Billy	Fahy			GlaxoSmithKline	Collegeville, PA, United States		FinnGen Study; Clinical Group / Task Force
Paola	Bronson			GlaxoSmithKline	Collegeville, PA, United States		FinnGen Study; Clinical Group / Task Force
Kirsi	Auro			GlaxoSmithKline	Espoo, Finland		FinnGen Study; Clinical Group / Task Force
David	Pulford			GlaxoSmithKline	Stevenage, United Kingdom		FinnGen Study; Clinical Group / Task Force
Sauli	Vuoti			Janssen-Cilag Oy	Espoo, Finland		FinnGen Study; Clinical Group / Task Force
Dermot	Reilly			Johnson & Johnson Innovative Medicine	Boston, MA, United States		FinnGen Study; Clinical Group / Task Force

Supplemental Online Content: Nonauthor Collaborators
*First name, last name, and suffix (if applicable) are required and will appear in PubMed.

***First Name and Middle Initial(s)**	***Last Name**	***Suffix (eg, Jr, III)**	Academic Degrees	Institution	Location (city, state/province, country)	Role or Contribution, eg, chair, principal investigator	Group (if more than 1 Group listed in the byline) and/or Subgroup (eg, Steering Committee)
Karen	He			Johnson & Johnson Innovative Medicine	Spring House, PA, United States		FinnGen Study; Clinical Group / Task Force
Ekaterina	Khramtsova			Johnson & Johnson Innovative Medicine	Spring House, PA, United States		FinnGen Study; Clinical Group / Task Force
Amy	Hart			Johnson & Johnson Innovative Medicine	Spring House, PA, United States		FinnGen Study; Clinical Group / Task Force
Meijian	Guan			Johnson & Johnson Innovative Medicine	Spring House, PA, United States		FinnGen Study; Clinical Group / Task Force
Alessandro	Porello			Johnson & Johnson Innovative Medicine	Spring House, PA, United States		FinnGen Study; Clinical Group / Task Force
P.	Dunnmon			Johnson & Johnson Innovative Medicine	Spring House, PA, United States		FinnGen Study; Clinical Group / Task Force
Sara	Gale			Johnson & Johnson Innovative Medicine	Spring House, PA, United States		FinnGen Study; Clinical Group / Task Force
Brice	Keyes			Johnson & Johnson Innovative Medicine	Spring House, PA, United States		FinnGen Study; Clinical Group / Task Force
John	Kwon			Johnson & Johnson Innovative Medicine	Spring House, PA, United States		FinnGen Study; Clinical Group / Task Force
Jonathan	Sherlock			Johnson & Johnson Innovative Medicine	Spring House, PA, United States		FinnGen Study; Clinical Group / Task Force

Supplemental Online Content: Nonauthor Collaborators
*First name, last name, and suffix (if applicable) are required and will appear in PubMed.

***First Name and Middle Initial(s)**	***Last Name**	***Suffix (eg, Jr, III)**	Academic Degrees	Institution	Location (city, state/province, country)	Role or Contribution, eg, chair, principal investigator	Group (if more than 1 Group listed in the byline) and/or Subgroup (eg, Steering Committee)
Matt	Loza			Johnson & Johnson Innovative Medicine	Spring House, PA, United States		FinnGen Study; Clinical Group / Task Force
Chris	Whelan			Johnson & Johnson Innovative Medicine	Spring House, PA, United States		FinnGen Study; Clinical Group / Task Force
W	Galpern			Johnson & Johnson Innovative Medicine	Spring House, PA, United States		FinnGen Study; Clinical Group / Task Force
Yanfei	Zhang			Johnson & Johnson Innovative Medicine	Spring House, PA, United States		FinnGen Study; Clinical Group / Task Force
Mona	Selej			Johnson & Johnson Innovative Medicine	Spring House, PA, United States		FinnGen Study; Clinical Group / Task Force
Abolfazl	Doostparast Torshizi			Johnson & Johnson Innovative Medicine	Spring House, PA, United States		FinnGen Study; Clinical Group / Task Force
Qingqin S	Li			Johnson & Johnson Innovative Medicine	Titusville, NJ, United States		FinnGen Study; Clinical Group / Task Force
Sahar	Mozzafari			Maze Therapeutics	San Francisco, CA, United States		FinnGen Study; Clinical Group / Task Force
Christopher	Deboever			Maze Therapeutics	San Francisco, CA, United States		FinnGen Study; Clinical Group / Task Force
Jason	Miller			Merck	Kenilworth, NJ, United States		FinnGen Study; Clinical Group / Task Force

Supplemental Online Content: Nonauthor Collaborators
*First name, last name, and suffix (if applicable) are required and will appear in PubMed.

***First Name and Middle Initial(s)**	***Last Name**	***Suffix (eg, Jr, III)**	Academic Degrees	Institution	Location (city, state/province, country)	Role or Contribution, eg, chair, principal investigator	Group (if more than 1 Group listed in the byline) and/or Subgroup (eg, Steering Committee)
Fabiana	Farias			Merck	Kenilworth, NJ, United States		FinnGen Study; Clinical Group / Task Force
Andrey	Loboda			Merck	Kenilworth, NJ, United States		FinnGen Study; Clinical Group / Task Force
Jorge	Del-aguila			Merck	Kenilworth, NJ, United States		FinnGen Study; Clinical Group / Task Force
Elisabeth	Vollmann			Merck	Kenilworth, NJ, United States		FinnGen Study; Clinical Group / Task Force
Jozsef	Karman			Merck	Kenilworth, NJ, United States		FinnGen Study; Clinical Group / Task Force
Julie	Fiore			Merck	Kenilworth, NJ, United States		FinnGen Study; Clinical Group / Task Force
Rajesh	Kamath			Merck	Kenilworth, NJ, United States		FinnGen Study; Clinical Group / Task Force
Andrei	Popescu			Merck	Kenilworth, NJ, United States		FinnGen Study; Clinical Group / Task Force
Delphine	Fagegaltier			Merck	Kenilworth, NJ, United States		FinnGen Study; Clinical Group / Task Force
Travis	Barr			Merck	Kenilworth, NJ, United States		FinnGen Study; Clinical Group / Task Force

Supplemental Online Content: Nonauthor Collaborators
*First name, last name, and suffix (if applicable) are required and will appear in PubMed.

***First Name and Middle Initial(s)**	***Last Name**	***Suffix (eg, Jr, III)**	Academic Degrees	Institution	Location (city, state/province, country)	Role or Contribution, eg, chair, principal investigator	Group (if more than 1 Group listed in the byline) and/or Subgroup (eg, Steering Committee)
Aristide	Merola			Merck	Kenilworth, NJ, United States		FinnGen Study; Clinical Group / Task Force
Oliver	Freeman			Merck	Kenilworth, NJ, United States		FinnGen Study; Clinical Group / Task Force
Simonne	Longerich			Merck	Kenilworth, NJ, United States		FinnGen Study; Clinical Group / Task Force
Enrico	Ferrero			Novartis Institutes for BioMedical Research	Cambridge, MA, United States		FinnGen Study; Clinical Group / Task Force
Nikos	Patsopoulos			Novartis Institutes for BioMedical Research	Cambridge, MA, United States		FinnGen Study; Clinical Group / Task Force
Nancy	Finkel			Novartis Institutes for BioMedical Research	Cambridge, MA, United States		FinnGen Study; Clinical Group / Task Force
Sabina	Pfister			Novartis Institutes for BioMedical Research	Cambridge, MA, United States		FinnGen Study; Clinical Group / Task Force
Shola	Richards			Novartis Institutes for BioMedical Research	Cambridge, MA, United States		FinnGen Study; Clinical Group / Task Force
Katherine	Mccauley			Novartis Institutes for BioMedical Research	Cambridge, MA, United States		FinnGen Study; Clinical Group / Task Force
Xiaobo	Xia			Novartis Institutes for BioMedical Research	Cambridge, MA, United States		FinnGen Study; Clinical Group / Task Force

Supplemental Online Content: Nonauthor Collaborators
*First name, last name, and suffix (if applicable) are required and will appear in PubMed.

***First Name and Middle Initial(s)**	***Last Name**	***Suffix (eg, Jr, III)**	Academic Degrees	Institution	Location (city, state/province, country)	Role or Contribution, eg, chair, principal investigator	Group (if more than 1 Group listed in the byline) and/or Subgroup (eg, Steering Committee)
Mike	Mendelson			Novartis Institutes for BioMedical Research	Cambridge, MA, United States		FinnGen Study; Clinical Group / Task Force
Majd	Mouded			Novartis	Basel, Switzerland		FinnGen Study; Clinical Group / Task Force
Debby	Ngo			Novartis	Basel, Switzerland		FinnGen Study; Clinical Group / Task Force
Kirsi	Kalpala			Pfizer	New York, NY, United States		FinnGen Study; Clinical Group / Task Force
Melissa	Miller			Pfizer	New York, NY, United States		FinnGen Study; Clinical Group / Task Force
Nan	Bing			Pfizer	New York, NY, United States		FinnGen Study; Clinical Group / Task Force
Jaakko	Parkkinen			Pfizer	New York, NY, United States		FinnGen Study; Clinical Group / Task Force
Heli	Lehtonen			Pfizer	New York, NY, United States		FinnGen Study; Clinical Group / Task Force
Stefan	McDonough			Pfizer	New York, NY, United States		FinnGen Study; Clinical Group / Task Force
Ying	Wu			Pfizer	New York, NY, United States		FinnGen Study; Clinical Group / Task Force

Supplemental Online Content: Nonauthor Collaborators
*First name, last name, and suffix (if applicable) are required and will appear in PubMed.

***First Name and Middle Initial(s)**	***Last Name**	***Suffix (eg, Jr, III)**	Academic Degrees	Institution	Location (city, state/province, country)	Role or Contribution, eg, chair, principal investigator	Group (if more than 1 Group listed in the byline) and/or Subgroup (eg, Steering Committee)
Erin	Macdonald-Dunlop			Pfizer	New York, NY, United States		FinnGen Study; Clinical Group / Task Force
Jessica	Chung			Pfizer	New York, NY, United States		FinnGen Study; Clinical Group / Task Force
Michael	McLean			Pfizer	New York, NY, United States		FinnGen Study; Clinical Group / Task Force
Joshua	Chiou			Pfizer	New York, NY, United States		FinnGen Study; Clinical Group / Task Force
Hye In	Kim			Pfizer	New York, NY, United States		FinnGen Study; Clinical Group / Task Force
Sivakumar	Pitchumani			Pfizer	New York, NY, United States		FinnGen Study; Clinical Group / Task Force
Sumedha	Jassal			Pfizer	New York, NY, United States		FinnGen Study; Clinical Group / Task Force
Madhurima	Saxena			Pfizer	New York, NY, United States		FinnGen Study; Clinical Group / Task Force
Catherine	O’Riordan			Translational Sciences, Sanofi R&D	Framingham, MA, USA		FinnGen Study; Clinical Group / Task Force
Samuel	Lessard			Translational Sciences, Sanofi R&D	Framingham, MA, USA		FinnGen Study; Clinical Group / Task Force

Supplemental Online Content: Nonauthor Collaborators
*First name, last name, and suffix (if applicable) are required and will appear in PubMed.

***First Name and Middle Initial(s)**	***Last Name**	***Suffix (eg, Jr, III)**	Academic Degrees	Institution	Location (city, state/province, country)	Role or Contribution, eg, chair, principal investigator	Group (if more than 1 Group listed in the byline) and/or Subgroup (eg, Steering Committee)
Suzanne	Jacobs			Translational Sciences, Sanofi R&D	Framingham, MA, USA		FinnGen Study; Clinical Group / Task Force
Hamid	Mattoo			Translational Sciences, Sanofi R&D	Framingham, MA, USA		FinnGen Study; Clinical Group / Task Force
David	Habiel			Translational Sciences, Sanofi R&D	Framingham, MA, USA		FinnGen Study; Clinical Group / Task Force
Guanling	Huan			Translational Sciences, Sanofi R&D	Framingham, MA, USA		FinnGen Study; Clinical Group / Task Force
Anu	Jalanko			Institute for Molecular Medicine Finland (FIMM), HiLIFE, University of Helsinki	Helsinki, Finland		FinnGen Study; FinnGen Teams (Administration)
Mervi	Aavikko			Institute for Molecular Medicine Finland (FIMM), HiLIFE, University of Helsinki	Helsinki, Finland		FinnGen Study; FinnGen Teams (Administration)
Helen	Cooper			Institute for Molecular Medicine Finland (FIMM), HiLIFE, University of Helsinki	Helsinki, Finland		FinnGen Study; FinnGen Teams (Administration)
Denise	Öller			Institute for Molecular Medicine Finland (FIMM), HiLIFE, University of Helsinki	Helsinki, Finland		FinnGen Study; FinnGen Teams (Administration)
Tarja	Laitinen			Institute for Molecular Medicine Finland (FIMM), HiLIFE, University of Helsinki	Helsinki, Finland		FinnGen Study; FinnGen Teams (Administration)
Rodos	Rodosthenous			Institute for Molecular Medicine Finland (FIMM), HiLIFE, University of Helsinki	Helsinki, Finland		FinnGen Study; FinnGen Teams (Administration)

Supplemental Online Content: Nonauthor Collaborators
*First name, last name, and suffix (if applicable) are required and will appear in PubMed.

***First Name and Middle Initial(s)**	***Last Name**	***Suffix (eg, Jr, III)**	Academic Degrees	Institution	Location (city, state/province, country)	Role or Contribution, eg, chair, principal investigator	Group (if more than 1 Group listed in the byline) and/or Subgroup (eg, Steering Committee)
Sofia	Kuitunen			University of Helsinki	Helsinki, Finland		FinnGen Study; FinnGen Teams (Administration)
Juha	Karjalainen			Institute for Molecular Medicine Finland (FIMM), HiLIFE, University of Helsinki	Helsinki, Finland		FinnGen Study; FinnGen Teams (Analysis)
Pietro	Della Briotta Parolo			Institute for Molecular Medicine Finland (FIMM), HiLIFE, University of Helsinki	Helsinki, Finland		FinnGen Study; FinnGen Teams (Analysis)
Arto	Lehisto			Institute for Molecular Medicine Finland (FIMM), HiLIFE, University of Helsinki	Helsinki, Finland		FinnGen Study; FinnGen Teams (Analysis)
Reza	Jabal			Institute for Molecular Medicine Finland (FIMM), HiLIFE, University of Helsinki	Helsinki, Finland		FinnGen Study; FinnGen Teams (Analysis)
Mutaamba	Maasha			Institute for Molecular Medicine Finland (FIMM), HiLIFE, University of Helsinki	Helsinki, Finland		FinnGen Study; FinnGen Teams (Analysis)
Sanni	Ruotsalainen			Institute for Molecular Medicine Finland (FIMM), HiLIFE, University of Helsinki	Helsinki, Finland		FinnGen Study; FinnGen Teams (Analysis)
Samuel	Jones			Institute for Molecular Medicine Finland (FIMM), HiLIFE, University of Helsinki	Helsinki, Finland		FinnGen Study; FinnGen Teams (Analysis)
Raymond	Walters			Institute for Molecular Medicine Finland (FIMM), HiLIFE, University of Helsinki	Helsinki, Finland		FinnGen Study; FinnGen Teams (Analysis)
Paavo	Häppölä			Institute for Molecular Medicine Finland (FIMM), HiLIFE, University of Helsinki	Helsinki, Finland		FinnGen Study; FinnGen Teams (Analysis)

Supplemental Online Content: Nonauthor Collaborators
*First name, last name, and suffix (if applicable) are required and will appear in PubMed.

***First Name and Middle Initial(s)**	***Last Name**	***Suffix (eg, Jr, III)**	Academic Degrees	Institution	Location (city, state/province, country)	Role or Contribution, eg, chair, principal investigator	Group (if more than 1 Group listed in the byline) and/or Subgroup (eg, Steering Committee)
Topi	Paavilainen			Institute for Molecular Medicine Finland (FIMM), HiLIFE, University of Helsinki	Helsinki, Finland		FinnGen Study; FinnGen Teams (Analysis)
L. Elisa	Lahtela			Institute for Molecular Medicine Finland (FIMM), HiLIFE, University of Helsinki	Helsinki, Finland		FinnGen Study; FinnGen Teams (Disease Task Forces)
Johanna	Paltta			Institute for Molecular Medicine Finland (FIMM), HiLIFE, University of Helsinki	Helsinki, Finland		FinnGen Study; FinnGen Teams (Disease Task Forces)
Juulia	Partanen			Institute for Molecular Medicine Finland (FIMM), HiLIFE, University of Helsinki	Helsinki, Finland		FinnGen Study; FinnGen Teams (Disease Task Forces)
Olli K.	Pietiläinen			Institute for Molecular Medicine Finland (FIMM), HiLIFE, University of Helsinki	Helsinki, Finland		FinnGen Study; FinnGen Teams (Disease Task Forces)
Veera	Timonen			Institute for Molecular Medicine Finland (FIMM), HiLIFE, University of Helsinki	Helsinki, Finland		FinnGen Study; FinnGen Teams (Disease Task Forces)
Mari	Kaunisto			Institute for Molecular Medicine Finland (FIMM), HiLIFE, University of Helsinki	Helsinki, Finland		FinnGen Study; FinnGen Teams (Communication)
Elina	Kilpeläinen			Institute for Molecular Medicine Finland (FIMM), HiLIFE, University of Helsinki	Helsinki, Finland		FinnGen Study; FinnGen Teams (Sandbox & Cloud Services)
Tianduanyi	Wang			Institute for Molecular Medicine Finland (FIMM), HiLIFE, University of Helsinki	Helsinki, Finland		FinnGen Study; FinnGen Teams (Sandbox & Cloud Services)

Supplemental Online Content: Nonauthor Collaborators
*First name, last name, and suffix (if applicable) are required and will appear in PubMed.

***First Name and Middle Initial(s)**	***Last Name**	***Suffix (eg, Jr, III)**	Academic Degrees	Institution	Location (city, state/province, country)	Role or Contribution, eg, chair, principal investigator	Group (if more than 1 Group listed in the byline) and/or Subgroup (eg, Steering Committee)
Timo P.	Sipilä			Institute for Molecular Medicine Finland (FIMM), HiLIFE, University of Helsinki	Helsinki, Finland		FinnGen Study; FinnGen Teams (Sandbox & Cloud Services)
Oluwaseun Alexander	Dada			Institute for Molecular Medicine Finland (FIMM), HiLIFE, University of Helsinki	Helsinki, Finland		FinnGen Study; FinnGen Teams (Sandbox & Cloud Services)
Awaisa	Ghazal			Institute for Molecular Medicine Finland (FIMM), HiLIFE, University of Helsinki	Helsinki, Finland		FinnGen Study; FinnGen Teams (Sandbox & Cloud Services)
Rigbe	Weldatsadik			Institute for Molecular Medicine Finland (FIMM), HiLIFE, University of Helsinki	Helsinki, Finland		FinnGen Study; FinnGen Teams (Sandbox & Cloud Services)
Jaska	Uimonen			Institute for Molecular Medicine Finland (FIMM), HiLIFE, University of Helsinki	Helsinki, Finland		FinnGen Study; FinnGen Teams (Sandbox & Cloud Services)
Kati	Donner			Institute for Molecular Medicine Finland (FIMM), HiLIFE, University of Helsinki	Helsinki, Finland		FinnGen Study; FinnGen Teams (Genotyping)
Anu	Loukola			Helsinki Biobank / Helsinki University and Hospital District of Helsinki and Uusimaa	Helsinki		FinnGen Study; FinnGen Teams (Sample Collection Coordination)
Päivi	Laiho			THL Biobank / Finnish Institute for Health and Welfare (THL)	Helsinki, Finland		FinnGen Study; FinnGen Teams (Sample Logistics)

Supplemental Online Content: Nonauthor Collaborators
*First name, last name, and suffix (if applicable) are required and will appear in PubMed.

***First Name and Middle Initial(s)**	***Last Name**	***Suffix (eg, Jr, III)**	Academic Degrees	Institution	Location (city, state/province, country)	Role or Contribution, eg, chair, principal investigator	Group (if more than 1 Group listed in the byline) and/or Subgroup (eg, Steering Committee)
Susanna	Lemmelä			Institute for Molecular Medicine Finland (FIMM), HiLIFE, University of Helsinki	Helsinki, Finland		FinnGen Study; FinnGen Teams (Register data and sample logistics)
Teemu	Paajanen			THL Biobank / Finnish Institute for Health and Welfare (THL)	Helsinki, Finland		FinnGen Study; FinnGen Teams (Register data and sample logistics)
Arto	Pietilä			THL Biobank / Finnish Institute for Health and Welfare (THL)	Helsinki, Finland		FinnGen Study; FinnGen Teams (Register data and sample logistics)
Aki	Havulinna			THL Biobank / Finnish Institute for Health and Welfare (THL)	Helsinki, Finland		FinnGen Study; FinnGen Teams (Register data and sample logistics)
Auli	Toivola			Institute for Molecular Medicine Finland (FIMM), HiLIFE, University of Helsinki	Helsinki, Finland		FinnGen Study; FinnGen Teams (Register data and sample logistics)
Kristina	Zguro			Institute for Molecular Medicine Finland (FIMM), HiLIFE, University of Helsinki	Helsinki, Finland		FinnGen Study; FinnGen Teams (Register data and sample logistics)
Mary Pat	Reeve			Institute for Molecular Medicine Finland (FIMM), HiLIFE, University of Helsinki	Helsinki, Finland		FinnGen Study; FinnGen Teams (Phenotype team)
Shanmukha Sampath	Padmanabhuni			Institute for Molecular Medicine Finland (FIMM), HiLIFE, University of Helsinki	Helsinki, Finland		FinnGen Study; FinnGen Teams (Phenotype team)

Supplemental Online Content: Nonauthor Collaborators
*First name, last name, and suffix (if applicable) are required and will appear in PubMed.

***First Name and Middle Initial(s)**	***Last Name**	***Suffix (eg, Jr, III)**	Academic Degrees	Institution	Location (city, state/province, country)	Role or Contribution, eg, chair, principal investigator	Group (if more than 1 Group listed in the byline) and/or Subgroup (eg, Steering Committee)
Harri	Siirtola			University of Tampere	Tampere, Finland		FinnGen Study; FinnGen Teams (Phenotype team)
Javier	Gracia-Tabuenca			University of Tampere	Tampere, Finland		FinnGen Study; FinnGen Teams (Phenotype team)
Marika	Kaakinen			Institute for Molecular Medicine Finland (FIMM), HiLIFE, University of Helsinki	Helsinki, Finland		FinnGen Study; FinnGen Teams (Phenotype team)
Shuang	Luo			Institute for Molecular Medicine Finland (FIMM), HiLIFE, University of Helsinki	Helsinki, Finland		FinnGen Study; FinnGen Teams (Phenotype team)
Vincent	Llorens			Institute for Molecular Medicine Finland (FIMM), HiLIFE, University of Helsinki	Helsinki, Finland		FinnGen Study; FinnGen Teams (Phenotype team)
Dawit	Yohannes			Institute for Molecular Medicine Finland (FIMM), HiLIFE, University of Helsinki	Helsinki, Finland		FinnGen Study; FinnGen Teams (Phenotype team)
Iina	Laak			Institute for Molecular Medicine Finland (FIMM), HiLIFE, University of Helsinki	Helsinki, Finland		FinnGen Study; FinnGen Teams (Data protection officer)
Pauli	Wihuri			Finnish Biobank Cooperative - FINBB			FinnGen Study; FinnGen Teams (FINBB - Finnish biobank cooperative)
Tom	Southerington			Finnish Biobank Cooperative - FINBB			FinnGen Study; FinnGen Teams (FINBB - Finnish biobank cooperative)

Supplemental Online Content: Nonauthor Collaborators
*First name, last name, and suffix (if applicable) are required and will appear in PubMed.

***First Name and Middle Initial(s)**	***Last Name**	***Suffix (eg, Jr, III)**	Academic Degrees	Institution	Location (city, state/province, country)	Role or Contribution, eg, chair, principal investigator	Group (if more than 1 Group listed in the byline) and/or Subgroup (eg, Steering Committee)
Meri	Lähteenmäki			Finnish Biobank Cooperative - FINBB			FinnGen Study; FinnGen Teams (FINBB - Finnish biobank cooperative)
