# Supplementary material for: Genome-Wide Association Study Meta-Analysis of Central Serous Chorioretinopathy
Source: JAMA Ophthalmol. 2026 Nov 5:e264427. Online ahead of print. doi: 10.1001/jamaophthalmol.2026.4427 (PMC13741192; doi:10.1001/jamaophthalmol.2026.4427)
Supplement: Supplementary file 3 [file jamaophthalmol-e264427-s004.pdf]

Supplemental Online Content: Nonauthor Collaborators
*First name, last name, and suffix (if applicable) are required and will appear in PubMed.

*Group Name(s): VA Million Veteran Program							
***First Name and Middle Initial(s)**	***Last Name**	***Suffix (eg, Jr, III)**	Academic Degrees	Institution	Location (city, state/province, country)	Role or Contribution, eg, chair, principal investigator	Group (if more than 1 Group listed in the byline) and/or Subgroup (eg, Steering Committee)
Sumitra	Muralidhar		Ph.D.	US Department of Veterans Affairs	Washington, DC	Program Director; Co-Chair	VA Million Veteran Program; MVP Program Office; MVP Steering Committee
Jennifer	Moser		Ph.D.	US Department of Veterans Affairs	Washington, DC	Associate Director, Scientific Programs	VA Million Veteran Program; MVP Program Office
Jennifer E.	Deen		B.S.	US Department of Veterans Affairs	Washington, DC	Associate Director, Cohort & Public Relations	VA Million Veteran Program; MVP Program Office
Philip S.	Tsao		Ph.D.	VA Palo Alto Health Care System	Palo Alto, CA	Co-Chair	VA Million Veteran Program; MVP Steering Committee; MVP Co-Principal Investigators
J. Michael	Gaziano		M.D., M.P.H.	VA Boston Healthcare System	Boston, MA		VA Million Veteran Program; MVP Steering Committee; MVP Co-Principal Investigators
Adriana	Hung		M.D., M.P.H.	VA Tennessee Valley Healthcare System	Nashville, TN		VA Million Veteran Program; MVP Steering Committee
Dave	Oslin		M.D.	Philadelphia VA Medical Center	Philadelphia, PA		VA Million Veteran Program; MVP Steering Committee
Deepak	Voora		M.D.	Durham VA Medical Center	Durham, NC		VA Million Veteran Program; MVP Steering Committee

Supplemental Online Content: Nonauthor Collaborators
*First name, last name, and suffix (if applicable) are required and will appear in PubMed.

***First Name and Middle Initial(s)**	***Last Name**	***Suffix (eg, Jr, III)**	Academic Degrees	Institution	Location (city, state/province, country)	Role or Contribution, eg, chair, principal investigator	Group (if more than 1 Group listed in the byline) and/or Subgroup (eg, Steering Committee)
Jessica V.	Brewer		M.P.H.	VA Boston Healthcare System	Boston, MA	Director, MVP Cohort Operations	VA Million Veteran Program; MVP Core Operations
Mary T.	Brophy		M.D., M.P.H.	VA Boston Healthcare System	Boston, MA	Director, VA Central Biorepository	VA Million Veteran Program; MVP Core Operations
Kelly	Cho		M.P.H, Ph.D.	VA Boston Healthcare System	Boston, MA	Director, MVP Phenomics	VA Million Veteran Program; MVP Core Operations
Lori	Churby		B.S.	VA Palo Alto Health Care System	Palo Alto, CA	Director, MVP Regulatory Affairs	VA Million Veteran Program; MVP Core Operations
Jacob T.	Kean		Ph.D.	VA Salt Lake City Health Care System	Salt Lake City, UT	Acting Director, VA Informatics and Computing Infrastructure (VINCI)	VA Million Veteran Program; MVP Core Operations
Saiju	Pyarajan		Ph.D.	VA Boston Healthcare System	Boston, MA	Director, Data and Computational Sciences	VA Million Veteran Program; MVP Core Operations
Robert	Ringer		Pharm.D.	New Mexico VA Health Care System	Albuquerque, NM	Director, VA Albuquerque Central Biorepository	VA Million Veteran Program; MVP Core Operations
Luis E.	Selva		Ph.D.	VA Boston Healthcare System	Boston, MA	Director, MVP Biorepository Coordination	VA Million Veteran Program; MVP Core Operations
Shahpoor (Alex)	Shayan		M.S.	VA Boston Healthcare System	Boston, MA	Director, MVP PRE Informatics	VA Million Veteran Program; MVP Core Operations

Supplemental Online Content: Nonauthor Collaborators
*First name, last name, and suffix (if applicable) are required and will appear in PubMed.

***First Name and Middle Initial(s)**	***Last Name**	***Suffix (eg, Jr, III)**	Academic Degrees	Institution	Location (city, state/province, country)	Role or Contribution, eg, chair, principal investigator	Group (if more than 1 Group listed in the byline) and/or Subgroup (eg, Steering Committee)
Brady	Stephens		M.S.	Canandaigua VA Medical Center	Canandaigua, NY	Principal Investigator, MVP Information Center	VA Million Veteran Program; MVP Core Operations
Stacey B.	Whitbourne		Ph.D.	VA Boston Healthcare System	Boston, MA	Director, MVP Cohort Development and Management	VA Million Veteran Program; MVP Core Operations
