# Supplementary material for: Genome-Wide Association Study Meta-Analysis of Central Serous Chorioretinopathy
Source: JAMA Ophthalmol. 2026 Nov 5:e264427. Online ahead of print. doi: 10.1001/jamaophthalmol.2026.4427 (PMC13741192; doi:10.1001/jamaophthalmol.2026.4427)
Supplement: Supplementary file 4 [file jamaophthalmol-e264427-s005.pdf]

Data Sharing Statement

Chen. Genome-Wide Association Study Meta-Analysis of Central Serous Chorioretinopathy. *JAMA Ophthalmol*. Published October 15, 2026. doi:10.1001/jamaophthalmol.2026.4427

Data
Data available: Yes
Data types: Data (not involving human participants)
How to access data: The complete summary statistics of the meta-analysis will be shared upon acceptance.
When available: With publication

Supporting Documents
Document types: None

Additional Information
Who can access the data: The summary statistics will be made available to the public.
Types of analyses: GWAS meta-analysis
Mechanisms of data availability: Public repository (Figshare)
